# Azithromycin Derivatives for Overcoming Antibacterial Resistance: Structural Modification and Structure–Activity Relationships

**DOI:** 10.3390/ijms27209110

**Published:** 2026-10-14

**Authors:** Kwanele Ngece, Athandwe M. Paca, Lisa V. Buwa-Komoreng, Blessings A. Aderibigbe, Vuyolwethu Khwaza

**Affiliations:** 1 Chemistry Discipline, Department of Chemical and Earth Sciences, Faculty of Science and Agriculture, University of Fort Hare, Alice 5700, South Africa; 201102901@ufh.ac.za; 2 Infectious Diseases and Medicinal Plants Research Niche Area, Botany Discipline, Department of Biotechnology and Biological Sciences, Faculty of Science and Agriculture, University of Fort Hare, Alice 5700, South Africa; lbuwa@ufh.ac.za; 3 Department of Chemistry, Physics, and Geosciences, Tarleton State University, Stephenville, TX 76402, USA; baderibigbe@tarleton.edu

**Keywords:** azithromycin, antimicrobial resistance, azithromycin derivatives, antibacterial agents, structural modification, structure–activity relationship, molecular hybridization

## Abstract

Antimicrobial resistance has significantly compromised the therapeutic efficacy of many antibiotics, highlighting the need for new agents capable of overcoming bacterial resistance. The discovery of antibiotics in the early 20th century contributed significantly to transforming modern medicine and saved countless lives. However, the alarming increase in antibiotic resistance now threatens these achievements. The misuse and overuse of antibiotics have driven the continuous evolution and adaptation of microorganisms, contributing to this growing global health challenge. Azithromycin (AZM), a widely used macrolide antibiotic with favorable pharmacokinetic properties, is increasingly affected by resistance mechanisms. A number of researchers have therefore designed and developed AZM derivatives with promising antibacterial properties. This review summarizes advances in the structural modification of AZM, focusing on side-chain modification, heterocyclic substitution, molecular hybridization, and their associated structure–activity relationships. Selected derivatives demonstrated enhanced activity against resistant bacterial pathogens compared with AZM, although activity varies according to substituent position, linker structure, and overall molecular properties. Nevertheless, the activity of many AZM derivatives against Gram-negative organisms remains limited, with restricted outer-membrane permeability and inadequate intracellular accumulation representing important barriers to achieving effective antibacterial activity. Overall, the structural modification of AZM represents a promising strategy for developing potential antibacterial agents, but further pharmacological and in vivo studies are required to establish their therapeutic potential.

## 1. Introduction

Antimicrobial resistance (AMR) has emerged as one of the most serious global public health challenges of the twenty-first century, threatening decades of progress in the treatment of bacterial infectious diseases [1,2,3]. The widespread emergence of multidrug-resistant (MDR) pathogens, including methicillin-resistant *Staphylococcus aureus* (MRSA), macrolide-resistant *Streptococcus pneumoniae*, vancomycin-resistant enterococci (VRE), and drug-resistant Gram-negative bacteria, has significantly reduced the effectiveness of many clinically available antibiotics [4,5,6]. It is estimated that antimicrobial resistance contributed to approximately 1.27 million deaths and was associated with up to 4.95 million deaths worldwide in 2019 [7]. If effective interventions are not implemented, antimicrobial resistance is projected to cause up to 10 million deaths annually by 2050 [8,9,10,11]. The continuous decline in the effectiveness of conventional antibiotics, together with the slow pace of new antibiotic discovery, highlights the urgent need for innovative antibacterial agents capable of overcoming existing resistance mechanisms while maintaining favorable pharmacokinetic and safety profiles.

Macrolide antibiotics remain one of the most successful classes of antibacterial agents due to their broad-spectrum activity, excellent tissue penetration, prolonged half-life, and safety characteristics [12,13,14]. Among them, azithromycin (AZM), a semisynthetic 15-membered azalide derived from erythromycin A, has become one of the most widely prescribed antibiotics for the treatment of respiratory tract infections [15,16], skin infections [17,18], sexually transmitted infections [19], gastrointestinal infections [20], ocular infections [21] and infections caused by atypical pathogens [22,23]. The insertion of a methyl-substituted nitrogen atom into the erythromycin lactone ring significantly improved acid stability, oral bioavailability, tissue distribution, and pharmacokinetic properties compared with its parent compound [24]. AZM exerts its antibacterial activity primarily through binding to domain V of the 23S rRNA within the 50S bacterial ribosomal subunit, thereby inhibiting peptide chain elongation and bacterial protein synthesis [12,25]. Despite these favorable properties, the clinical efficacy of AZM has been progressively compromised by the rapid emergence of resistance in various bacterial strains (Table 1). These resistance mechanisms significantly reduce the affinity of AZM for its molecular target and contribute to therapeutic failure. Moreover, prolonged and inappropriate use of macrolides has accelerated the dissemination of resistant bacterial strains [26], further emphasizing the need for next-generation macrolide antibiotics.

Medicinal chemists have therefore currently focused on the rational structural modification of AZM to restore antibacterial potency and overcome established resistance mechanisms. The unique molecular architecture of AZM provides multiple chemically accessible positions for derivatization, including the C3, C4″, C9a, C11, and C12 positions, as well as modifications of the desosamine and cladinose sugar moieties. These structural alterations have enabled the development of different classes of azithromycin derivatives.

Among the various approaches used to modify AZM, molecular hybridization has emerged as a prominent strategy for developing derivatives with enhanced antibacterial activity [43]. Potentially, these new hybrids offer high potential to reduce the risk of drug–drug interaction, improve pharmacokinetic properties, minimize the toxicity of the parent drug, and enhance patient compliance [44,45]. Hybrid compounds are designed to maintain their activities by merging two or more bioactive compounds or their pharmacophoric subunits into a new molecular structure with a dual mode of action. The concept of design and preparation of hybrid molecules is achieved using two strategies: (1) combining drug pharmacophoric moieties and (2) combining two or more entire drugs. The first approach involves merging two functional groups from different drugs, acting through the same or different mechanisms of action. In the second approach, two or more bioactive agents are connected either directly or indirectly via a spacer or linker [46,47]. A molecular linker/spacer is typically used to attach the constituent agents via a covalent bond. The covalent bond can be cleavable (e.g., esters, amides, carbamates, or disulfide bonds) or non-cleavable [48]. It is expected that the cleavable linker will be bio-transformed upon arrival at the target site of action. On the other hand, a non-cleavable linker maintains its structure throughout its time in the body/host. The cleavable linker is a pro-drug strategy. The non-cleavable linker is a hybrid drug approach [49].

Several reviews have discussed macrolide antibiotics [4,50,51], the pharmacological properties of AZM [52,53,54,55], and selected mechanisms of macrolide resistance [56,57,58]. However, a focused analysis integrating recent structural modifications of AZM with their antibacterial activity, resistance-breaking properties, and emerging structure–activity relationship (SAR) trends remains limited. Therefore, this review focuses on advances in the structural modification of AZM, with particular emphasis on how modification site, substituent identity, linker architecture, molecular hybridization, and physicochemical properties influence antibacterial activity and activity against resistant bacterial strains.

## 2. Literature Search Strategy

A focused literature search was conducted to identify published studies relevant to the structural modification, synthesis, antibacterial evaluation, and SARs of AZM derivatives. The search mainly covered literature published between 2020 and 2026, with particular emphasis on recent studies describing structural strategies aimed at modifying the AZM scaffold to improve antibacterial activity or address resistance.

A thorough literature search was performed using search engines or databases such as PubMed, ScienceDirect, Scopus, Web of Science, and Google Scholar. The search covered publications with the following combinations of keywords and Boolean operators such as “azithromycin” AND “derivatives”; “azithromycin” AND “structural modification”; “azithromycin derivatives” AND “antibacterial activity”; “azithromycin” AND “structure–activity relationship”; “azithromycin” AND “macrolide resistance”; “azithromycin” AND “molecular hybridization”; “azithromycin” AND “heterocyclic derivatives”; and “azithromycin” AND “resistant bacteria.” Additional relevant studies were identified by screening the reference lists of selected publications.

Studies were selected based on their relevance to the objectives and scope of this narrative review [59]. Particular attention was given to publications reporting the synthesis or structural modification of AZM or AZM-based derivatives, experimental antibacterial activity, activity against susceptible or resistant bacterial strains, and/or SAR information linking specific structural modifications to antibacterial properties. Publications focusing exclusively on non-antibacterial applications of AZM, clinical use of unmodified AZM without structural modification, unrelated biological activities, or studies that did not provide sufficient information on the investigated compounds were generally not considered.

## 3. Azithromycin Overview

**Figure 1:**
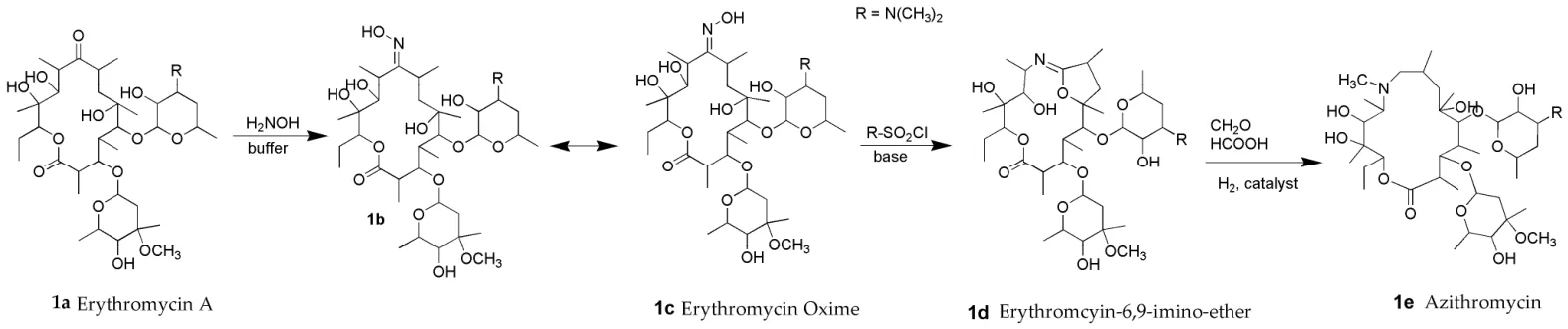
Synthetic development and discovery of AZM from erythromycin A [65].

Erythromycin, discovered in 1952 from *Streptomyces erythraeus* (now *Saccharopolyspora erythraea*), was previously an important macrolide antibiotic; however, its clinical use was limited by acid instability, low oral bioavailability, gastrointestinal intolerance, and an unpleasant taste [60,61]. The development of azalides through the Beckmann rearrangement of erythromycin A oxime provided a 15-membered macrolide scaffold with improved acid stability and enhanced activity against Gram-negative bacteria [26,62,63]. Subsequent reductive methylation of 9-dihydro-9-deoxo-9a-aza-9a-homoerythromycin A produced azithromycin (AZM, **1e**), a clinically important azalide antibiotic (Figure 1) [64].

AZM is a 15-membered macrolide characterized by an expanded aglycone ring containing a methyl-substituted nitrogen at C-9, together with the desosamine and cladinose sugar residues [65,66,67]. These structural features contribute to its favorable pharmacokinetic properties, including extensive tissue distribution, cellular accumulation, and a prolonged terminal half-life [66]. Like other macrolides, AZM exerts its antibacterial activity primarily through reversible binding to the bacterial 50S ribosomal subunit, specifically the 23S rRNA, thereby interfering with protein synthesis [12,68]. Despite its clinical utility, the emergence of antimicrobial resistance has created a need for AZM derivatives with improved antibacterial properties.

**Figure 2:**
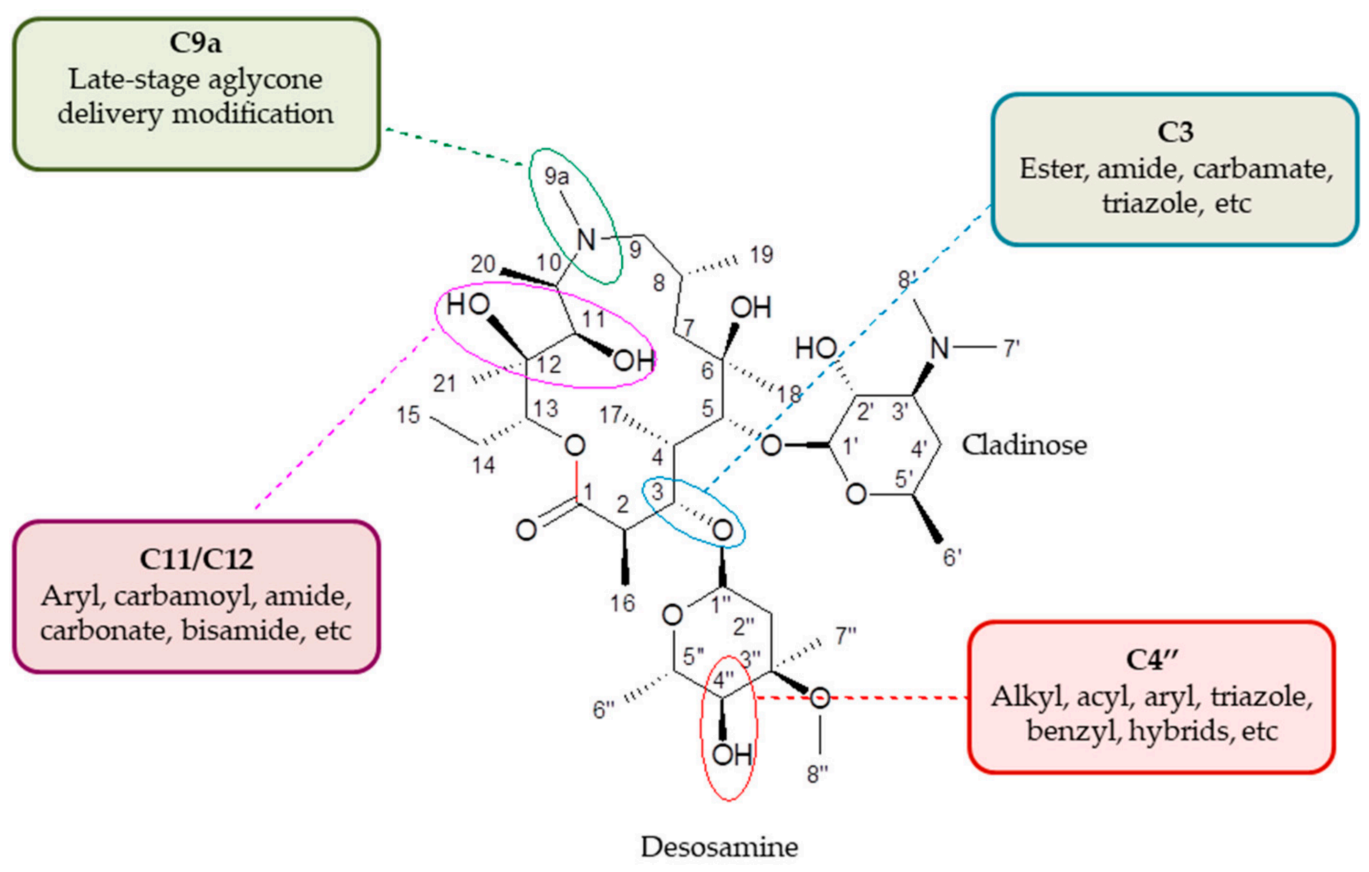
Chemical structure of AZM showing the 15-membered azalide scaffold and key sites for structural modification on the AZM scaffold [69,70].

The AZM scaffold contains several chemically accessible sites that can be exploited for medicinal-chemistry optimization while retaining the essential macrolide pharmacophore (Figure 2). Reported modification strategies include functionalization at C-3, C-4″, C-9a, C-11, and C-12; modification of the desosamine and cladinose sugar residues; incorporation of heterocyclic substituents; and molecular hybridization with other antibacterial pharmacophores. These structural modification strategies and their effects on antibacterial activity and structure–activity relationships are discussed in the following sections.

## 4. Structural Modification of Azithromycin and Antibacterial Activity

Bacterial infections can lead to prolonged hospitalization, increased mortality, and morbidity. Because molecular hybrids may combine two pharmacophores and potentially exhibit complementary or dual mechanisms of action, they have attracted considerable interest in medicinal chemistry. This section summarizes AZM-based derivatives reported to exhibit antibacterial activity against selected bacterial pathogens.

**Figure 3:**
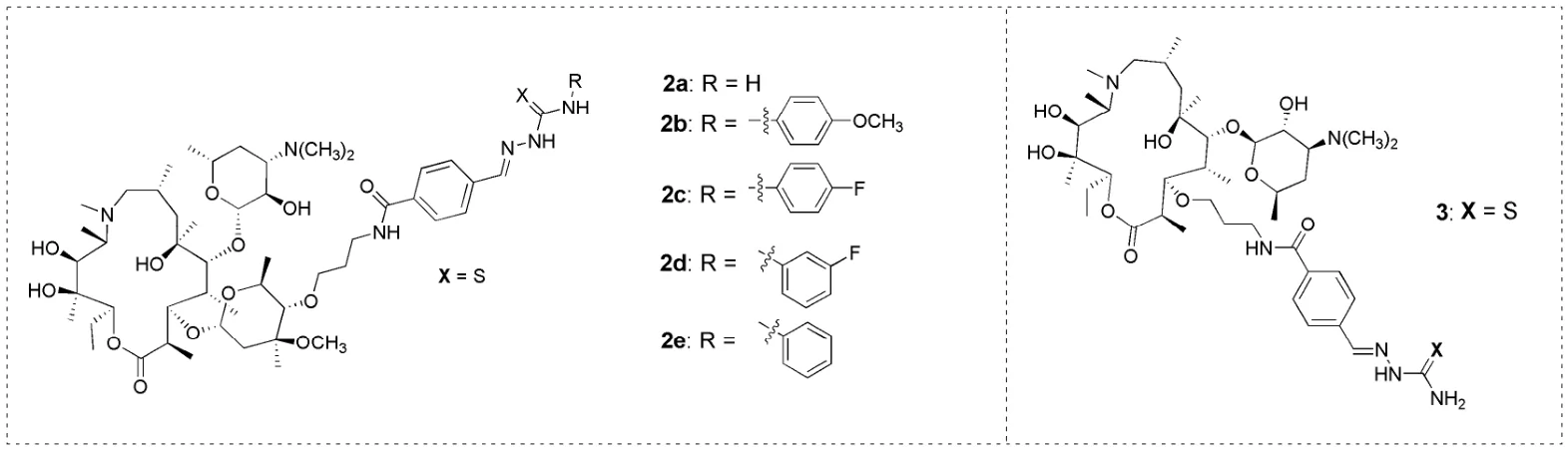
Chemical structures of thiosemicarbazone-based AZM conjugates **2a**–**2e** and **3** [68].

Grgicevic and colleagues [68] synthesized antimicrobial thiosemicarbazone-based AZM conjugates (Figure 3) and evaluated their biological activity against selected Gram-positive and Gram-negative bacterial strains. Compounds **2a**–**2e** were obtained by amidation of the AZM-derived amine with thiosemicarbazone acids using HATU/DIPEA in DCM at room temperature. Compound **3** was similarly synthesized by coupling the C-3 aminopropyl AZM intermediate with thiosemicarbazone acids under HATU/DIPEA-mediated conditions. All compounds showed excellent activity against *S. pneumoniae* (Table 2). Moreover, compounds **2a**–**2e** and **3** showed up to 32-fold greater activity against efflux-resistant *S. pneumoniae* (M phenotype) than AZM, with MIC values ranging from <0.125 to 2 µg/mL. Good activity against *Streptococcus pyogenes* was also observed. All compounds displayed somewhat lower potency against susceptible *S. aureus* than AZM. However, derivatives **2a**–**2e** exhibited good activity against a resistant *S. aureus* strain, whereas AZM showed no significant activity against this strain. Compound **3** also showed excellent activity against *Enterococcus faecalis* (MIC < 0.125 µg/mL). The 4″-derivatives (**2a**–**2e** and **3**) displayed moderate activity against Gram-negative *Haemophilus influenzae*. The authors attributed the enhanced activity to the proposed dual mode of action of the macrozones [68].

**Figure 4:**
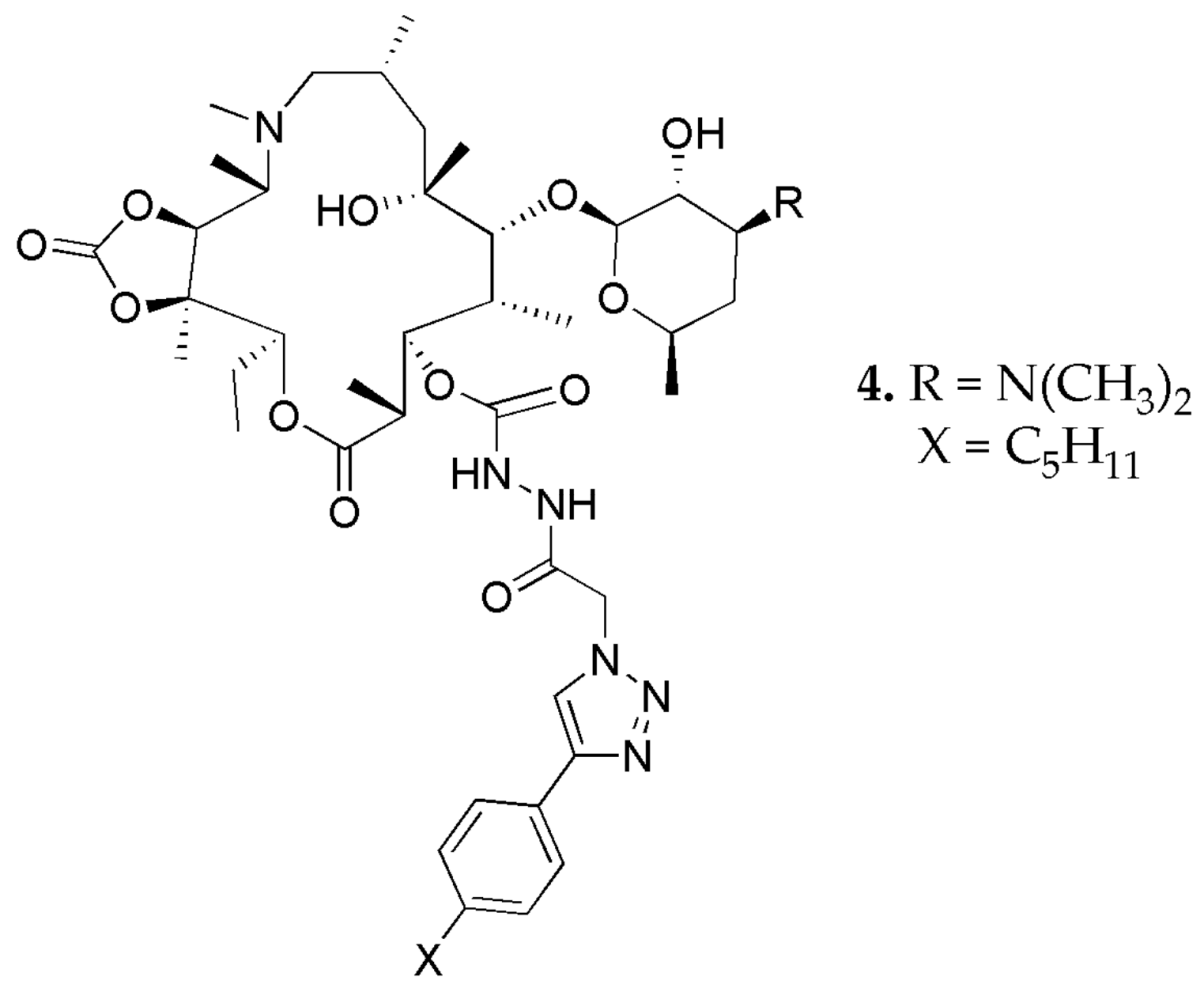
Chemical structure of the 3-O-substituted 15-membered azalide derivative **4** [71].

Qin and colleagues synthesized a novel 3-O-substituted 15-membered azalide, compound **4**, bearing a 1,2,3-triazole side chain (Figure 4), and evaluated its antibacterial activity against selected strains [71]. The synthesis of compound **4** began with conversion of AZM to 3-O-decladinosyl azithromycin, followed by acetylation, acylation, hydrazinolysis, and sequential amine acylation/azidation to afford the C-3 azide derivative. A CuI-catalyzed click reaction of the C-3 azide derivative with phenylacetylene or substituted phenylacetylenes produced intermediates that were selectively deprotected at the 2′-O-acetyl group in methanol to give the target 3-O-substituted 15-membered azalide **4**. Compound **4** showed activity comparable to AZM against *E. faecalis* with an MIC value of 4 µg/mL for both compounds (Table 3). Compound **4** showed MIC values of 8 µg/mL against *S. aureus* ATCC25923, and *Bacillus subtilis* ATCC9372 while an MIC of 4 µg/mL was observed against *E. faecalis*. Notably, Compound **4** was more active than AZM against the clinical isolates of *Enterococcus faecium*, *S. aureus* ATCC43300, and *S. aureus* PR. Against the clinical *Enterococcus faecium* isolate, compound **4** exhibited an MIC value of 8 µg/mL compared with 256 µg/mL for AZM, representing a 32-fold lower MIC. Against *S. aureus* ATCC43300 and *S. aureus* PR, Compound **4** showed MICs of 16 µg/mL, whereas AZM exhibited MICs of >256 µg/mL, corresponding to at least a 16-fold lower MIC for Compound **4**. The SAR of compound **4** can be summarized as follows: the derivative bearing the longest alkyl substituent, a pentyl group, on the 1,2,3-triazole side chain generally showed potent antibacterial activity against *E. faecium*, *S. aureus* ATCC 43300, and *S. aureus* PR. Molecular docking indicated that the core structures of compound **4** and AZM largely overlapped, whereas the pentyl group extended toward the A752 base of the *E. coli* ribosome. The authors proposed that compound **4** may interact with A752 of 23S rRNA through hydrophobic or electrostatic interactions, which could contribute to its activity against resistant bacteria. Compound **4** was also selected for bactericidal kinetic studies, which indicated time- and concentration-dependent activity [71].

**Figure 5:**
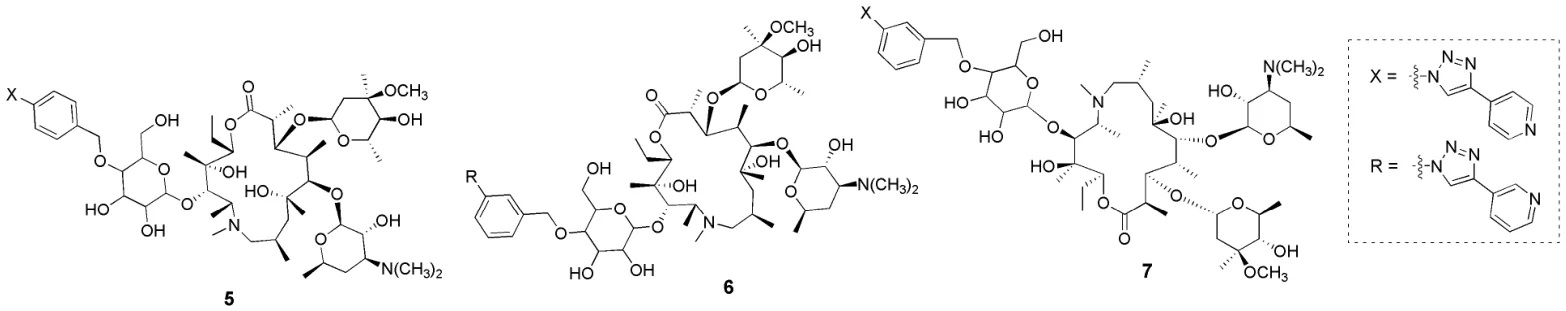
Chemical structures of azithromycin derivatives **5**–**7** evaluated against non-tuberculous mycobacterial strains [72,73].

Isozaki synthesized AZM derivatives **6** and **7** and evaluated their activity against non-tuberculous mycobacteria [72]. These derivatives were synthesized through a three-step sequence: (i) a late-stage BMAD reaction using a p-N3-OBn glucose donor, (ii) deprotection, and (iii) Huisgen cycloaddition. Derivatives **6** and **7** contained substituents extending from the *meta* position of the benzyl group at O_4_ of the glucose moiety. In this study, compounds **6** and **7** were compared with the previously reported AZM derivative **5** (Figure 5, Table 4) [73]. AZM and telithromycin (TEL) were used as controls. AZM displayed antibacterial activity with MIC values of ≤1 µg/mL against wild-type MAC but was not significantly active against macrolide-resistant MAC strains (MIC > 128 µg/mL). In contrast, TEL did not demonstrate significant activity against macrolide-resistant *Mycobacterium intracellulare* (MIC = 32 µg/mL) but showed activity against both wild-type MAC (MIC ≤ 1 µg/mL) and macrolide-resistant *M. avium* (MIC = 4 µg/mL). Compounds **6** and **7** bearing *meta*-substituted benzyl moieties at O_4_ of glucose were inactive against macrolide-resistant *M. intracellulare* (MICs ≥ 64 µg/mL). In contrast, compounds **6** and **7** exhibited MICs ≤ 16 µg/mL against macrolide-resistant *M. avium*. Compound **6**, bearing a 3-pyridyl moiety, exhibited an MIC of 4 µg/mL against macrolide-resistant *M. avium*, comparable to TEL. For wild-type *M. avium* and *M. intracellulare*, compound **7** showed lower MIC values than derivative **5** (0.0625 µg/mL for both strains versus 0.25 and 0.125 µg/mL, respectively). These findings suggest that the position of the triazole-containing substituent influences activity against wild-type strains. Compound **6** displayed an MIC of 0.0312 µg/mL against *M. intracellulare*, which was fourfold lower than that of TEL (0.125 µg/mL). Similarly, compound **7**, bearing a 4-pyridyl moiety, exhibited an MIC of 0.0625 µg/mL against *M. avium*, representing a 16-fold lower MIC than TEL (1 µg/mL). Compound **7** also showed an MIC of 0.0625 µg/mL against *M. intracellulare*. The meta-substituted benzyl moiety in compounds **6** and **7** was associated with enhanced activity against wild-type MAC, whereas the para-substituted benzyl group in compound **5** was associated with activity against macrolide-resistant strains [72].

**Figure 6:**
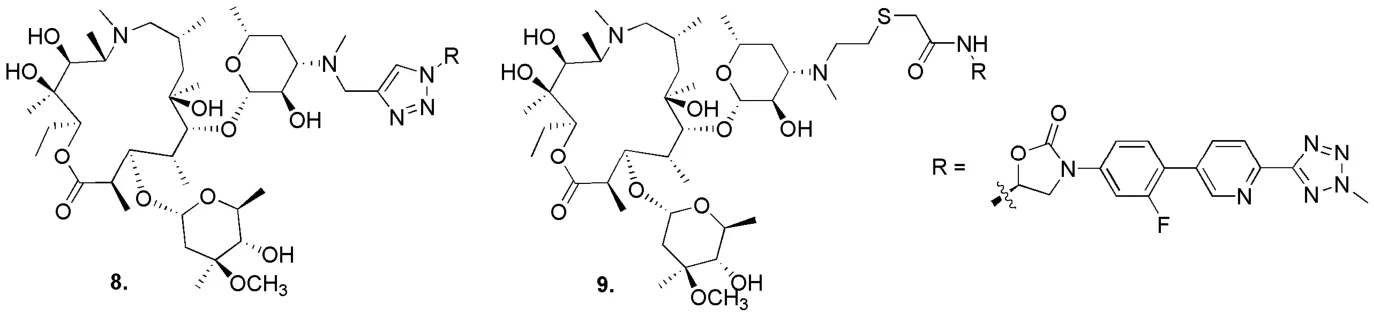
Chemical structures of azithromycin–tedizolid hybrid compounds **8** and **9** [74].

Yeon and colleagues synthesized compounds **8** and **9** (Figure 6), AZM–tedizolid (TDZ) hybrids designed to target the bacterial ribosome, and evaluated them against selected bacterial strains [74]. TDZ was first converted to the azide by methanesulfonylation followed by sodium azide substitution. Subsequent Cu-catalyzed azide–alkyne cycloaddition with an alkyne afforded hybrid **8**. For hybrid **9**, the azide was reduced to a primary amine and acylated with chloroacetyl chloride, followed by reaction with 2-mercaptoethanol and methanesulfonylation. The resulting product was coupled with AZM to produce the extended-linker AZM–TDZ hybrid **9**. As shown in Table 5, compounds **8** and **9** inhibited the growth of *S. aureus* with MICs = 0.5 and 0.25 µg/mL, respectively, compared with 2–32 µg/mL for AZM. To investigate the effect of the linker on binding, cryo-EM structures of compounds **8** and **9** in complex with the *E. coli* ribosome were determined. When aligned with the AZM macrocycle, the TDZ portion of hybrid **8** was displaced relative to the native TDZ binding site, potentially because of the rigid triazole linker. In the cryo-EM structure of **9**, strong electron density was observed in one conformation that positioned the TDZ portion appropriately. Compound **9** was active against all *erm-*mediated resistant strains tested, including inducible and constitutive phenotypes. It also retained activity against strains carrying mutations in L4 and L22 ribosomal proteins and 23S rRNA mutations (A2058G/A2059G), which typically confer macrolide resistance. Compound **9** exhibited activity comparable to TDZ; however, Cfr-mediated methylation of A2503 in *Staphylococci* induced resistance to **9** but not to TDZ. Compound **9** retained activity against almost all strains tested, including those with constitutive resistance to macrolides and oxazolidinones. In most strains, its MICs were 2–8-fold higher than those of TDZ. Compared with linezolid, compound **9** was either equally active or more active against all strains tested. The effects of compounds **8** and **9** on protein synthesis in human cells were also evaluated, demonstrating modulation of both cytosolic and mitochondrial protein synthesis in a manner distinct from the parent drugs. This study provides a framework for the design, synthesis, and evaluation of hybrid molecules targeting the bacterial ribosome [74].

**Figure 7:**
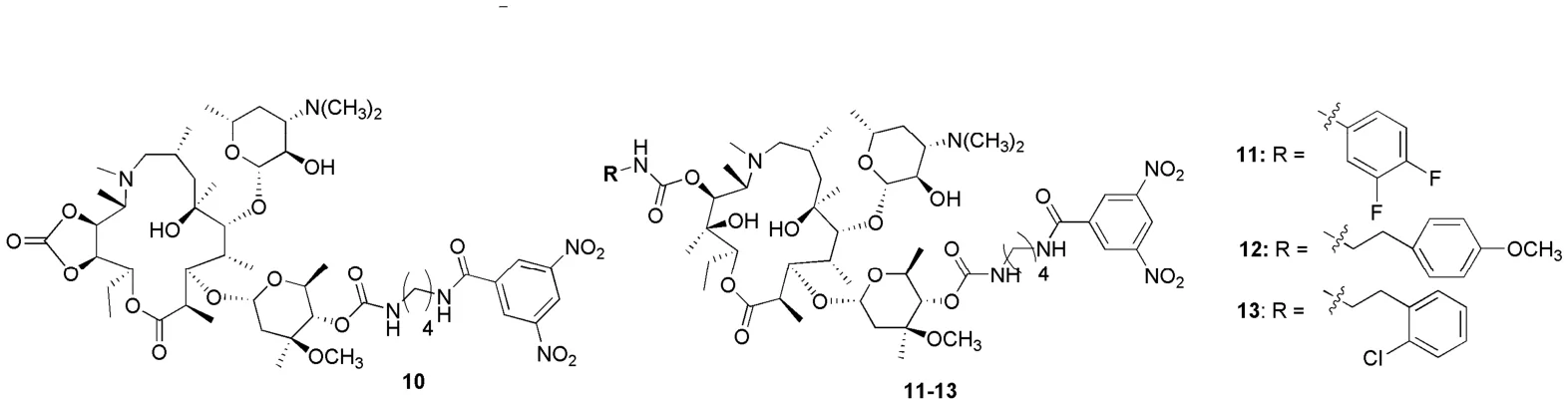
Chemical structures of precursor **10** and the **11**, 4″-disubstituted azithromycin derivatives **11**–**13** [75].

Li et al. synthesized novel 11,4″-disubstituted AZM analogues **11**–**13** and evaluated their antibacterial activity [75]. AZM was sequentially acetylated at the 2′-hydroxyl group and converted at the 4″-hydroxyl group to an acyl imidazolide, followed by cyclization with different diamines to afford 11,12-cyclic carbonate intermediates and subsequent coupling/deprotection to give key intermediate 10. Intermediate 10 was then coupled with the corresponding primary amines in the presence of pyridine hydrochloride at room temperature to produce the 11,4″-disubstituted AZM analogues 11–13 in 60–80% yields. Compounds **11**–**13** (Figure 7, Table 6), which contained the same terminal 3,5-dinitrophenyl group on their C-11 side chains and were synthesized from the same precursor **10**, demonstrated strong activity against *S. pneumoniae* ATCC 49619, *S. aureus* ATCC 25923, *Salmonella typhimurium* TA102, *S. pneumoniae* B1, *S. pneumoniae* A22072, and *S. pneumoniae* AB11 (Table 6). The MIC values were 0.03, 0.03, and 0.03 µg/mL against *S. pneumoniae* ATCC 49619; 0.25, 0.25, and 0.12 µg/mL against *S. aureus* ATCC 25923; 0.5, 0.5, and 0.25 µg/mL against *S. typhimurium* TA102; 0.25, 0.25, and 0.25 µg/mL against *S. pneumoniae* B1; 0.03, 0.03, and 0.03 µg/mL against *S. pneumoniae* A22072; and 2, 2, and 2 µg/mL against *S. pneumoniae* AB11, respectively. Compounds **11**–**13** were 512-, 133-, and 128-fold more active than AZM against erythromycin-resistant *S. pneumoniae* B1, *S. pneumoniae* A22072, and *S. pneumoniae* AB11, respectively. Compounds **11**–**13** showed MICs of 0.03 µg/mL against erythromycin-susceptible *S. pneumoniae* ATCC 49619, whereas compound **13** showed an MIC of 0.12 µg/mL against erythromycin-susceptible *S. aureus* ATCC 25923. Among the tested erythromycin-resistant strains, compounds **11**–**13** showed MICs of 0.25 µg/mL against *S. pneumoniae* B1, 2 µg/mL against *S. pneumoniae* AB11 and 0.03 µg/mL against *S. pneumoniae* A22072. These findings indicate that the introduction of C-11 aryl-carbamoyl side chains together with elongated C-4″ bisamide side chains can enhance activity against selected Gram-positive bacteria [75].

**Figure 8:**
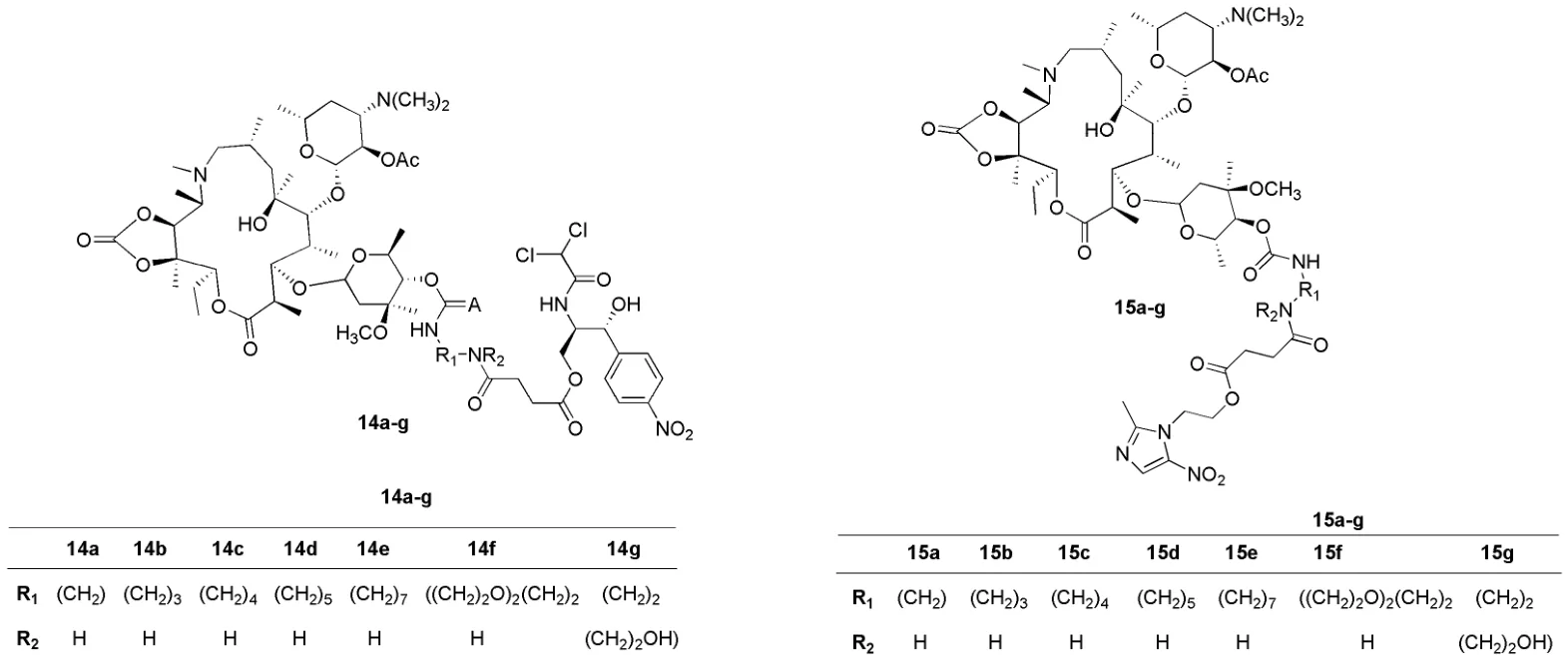
Chemical structures of azithromycin–chloramphenicol hybrids **14a**–**g** and azithromycin–metronidazole hybrids **15a**–**g** [76].

Volynkina and colleagues synthesized hybrid molecules of AZM with chloramphenicol (CLM) (**14a**–**g**) and metronidazole (MNZ) (**15a**–**g**) (Figure 8) and evaluated their antibacterial activity against selected bacterial strains [76]. AZM was first converted to the key intermediate by 2′-O-acetylation, followed by CDI-mediated carbamoylation at the 4″ position and formation of the 11,12-cyclic carbonate. The obtained intermediate was then reacted with various diamines to introduce different linker structures, and the resulting crude carbamates were directly coupled with chloramphenicol or metronidazole hemisuccinate using PyBOP, affording the AZM–CLM (**14a**–**g**) and AZM–MNZ (**15a**–**g**) hybrid series. The AZM-CLM hybrids (**14a**–**g**) exhibited submicromolar antibacterial activity against Gram-positive *S. pneumoniae* ATCC 49619 (Table 7). Conjugation of AZM with CLM slightly attenuated potency compared with AZM; however, the MIC values of all new compounds **14a**–**g** were lower than those of CLM. Introduction of a more flexible diethylene glycol fragment in **14f** contributed to the antibacterial activity of the hybrid compounds. Conjugation of AZM with MNZ (**15a**–**g**) generally resulted in decreased antibacterial activity compared with the parent antibiotics. However, compound **15a** exhibited enhanced activity against *Clostridium sporogenes* ATCC 19404, with an MIC of 0.25 µg/mL, compared with 4 µg/mL for AZM. *S. pneumoniae* was the most susceptible organism to the AZM-MNZ hybrids, with activity comparable to AZM but significantly higher than that of MNZ. In contrast, **15a**–**g** were less active against *S. agalactiae* 1Cp and *S. aureus* ATCC 29213. For derivatives **15a**–**g**, a consistent relationship was observed between linker structure and antibacterial activity, with the 2-(2-(2-aminoethoxy)ethoxy)ethane linker associated with the highest activity in compounds **14f** and **15f**. The AZM-MNZ hybrid **15f** was fourfold more active than the AZM-CLM derivative **14f** against *S. aureus*. Overall, AZM-MNZ hybrids **15a**–**g** were similar to or slightly more active than AZM-CLM hybrids **14a**–**g**. Nevertheless, all AZM hybrid compounds were less active than AZM, possibly due to reduced cellular penetration [76].

**Figure 9:**
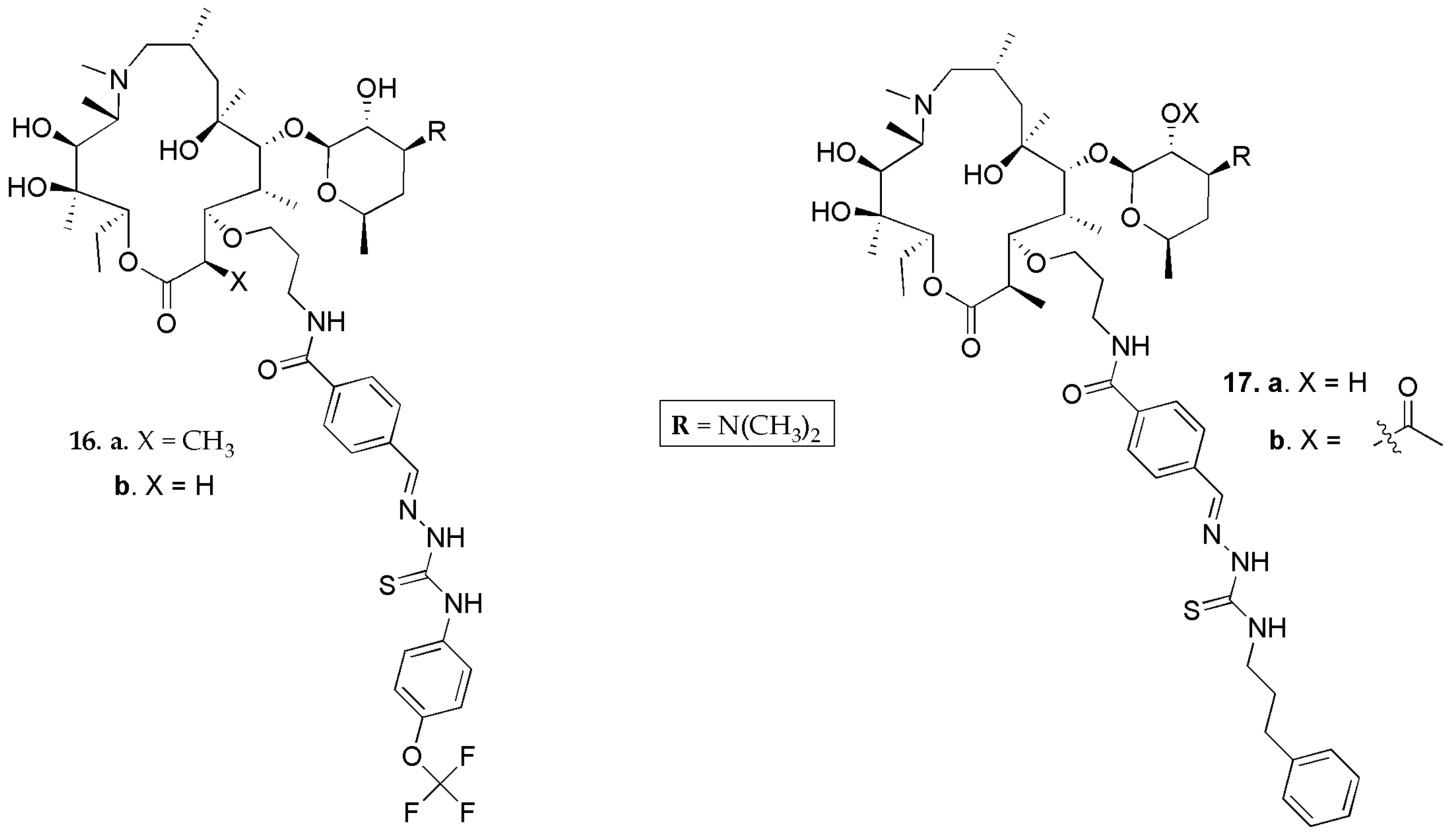
Chemical structures of the azithromycin–thiosemicarbazone macrozones (**16a**, **17a**) and their related stereoisomeric derivatives (**16b**, **17b**) [65].

Habinovec and colleagues synthesized AZM–thiosemicarbazone conjugates (**16a**,**b** and **17a**,**b**) (Figure 9) and evaluated their antibacterial activity against selected pathogens [65]. The main compounds **16a** and **17a**, together with their diastereomers and derivatives **16b** and **17b**, were synthesized according to a method described by Grgičević et al. [68]. Macrozone **16a** showed somewhat greater overall antibacterial activity than **17b** (Table 8). Compared with AZM, both compounds showed improved potency against constitutively resistant strains and similar or greater activity against efflux-resistant Gram-positive strains including *S. pneumoniae*, *S. pyogenes*, and *S. aureus*. Biological activity also varied among epimers. Compared with **16a**, compound **16b** was less active against efflux-resistant *S. pneumoniae* and *S. pyogenes* but more active against constitutive-resistant *S. aureus*. Isomer **17b** exhibited lower activity than the main compound **17a**. These findings demonstrate that relatively small conformational differences among epimeric structures can influence biological activity and may be important for further derivatization of macrolide scaffolds [65].

**Figure 10:**
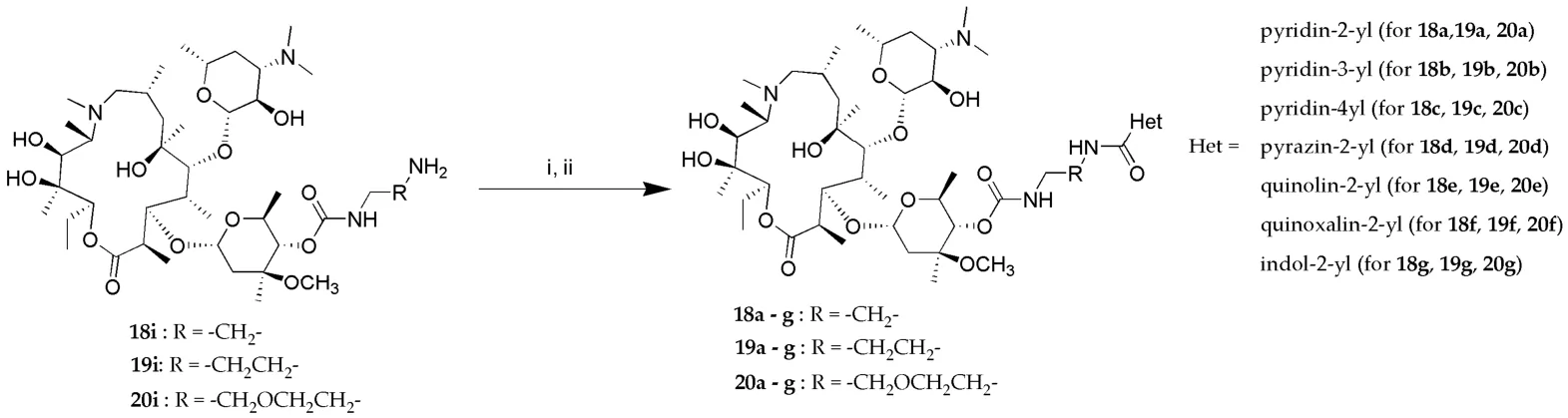
Synthesis of azithromycin derivatives **18a**–**g**, **19a**–**g**, and **20a**–**g.** Reagents and conditions: (i) Carboxylic acid, PyBOP, DIPEA, DMF, rt, 1 h; (ii) MeOH, 40 °C, 48 h [77].

Martynov et al. synthesized 4″-modified AZM derivatives (**18a**–**g**, **19a**–**g**, and **20a**–**g**) bearing nitrogen-containing heterocycles with dual inhibitory activity and evaluated them against selected Gram-positive and Gram-negative bacterial strains [77]. As shown in Figure 10, substituted 4″-modified AZM carbamates containing ethylene (**18i**), propylene (**19i**), or oxybis(ethane) (**20i**) linkers were used as intermediates for conjugation. The primary amino groups were acylated with various aza-heterocyclic carboxylic acids using PyBOP/DIPEA in DMF, followed by silica gel purification and removal of the 2′-O-acetyl group with methanol. This afforded three series of AZM–heterocycle conjugates, **18a**–**g**, **19a**–**g**, and **20a**–**g**, with different linker lengths. All conjugates, except for **18c**, **19c**, and **20c**, which contained an isonicotinamide fragment, demonstrated good activity against the macrolide-sensitive strain *S. pneumoniae* ATCC 49619. However, only certain derivatives, specifically those containing fused heterocyclic moieties such as quinoline, quinoxaline, and indole (**18e**–**g**, **19e**–**g**, and **20e**–**g**) or picolinic acid (**18a**, **19a**, and **20a**), demonstrated notable activity against other susceptible strains, including *S. aureus* ATCC 29213 and *E. faecalis* ATCC 29212 (Table 9). Against the representative Gram-negative organism *E. coli*, the synthesized derivatives generally exhibited elevated MIC values, consistent with reduced bacterial penetration; however, this explanation was inferred from MIC data rather than directly demonstrated by uptake or permeability experiments [78,79,80]. SAR analysis suggested that the 3-aminopropylcarbamate linker in series **19a**–**g** generally conferred superior antibacterial activity than the alternative linkers. Derivatives **18f**, **19a**, **19e**, **19f**, and **19g** were also evaluated against macrolide-resistant *S. pneumoniae* strains, including *S. pneumoniae* B-24 (*met*-positive) and *S. pneumoniae* B-1 (*erm*-positive), representing active efflux and ribosomal target methylation, respectively. All selected derivatives inhibited growth of both resistant strains. These findings suggest that the introduced heterocyclic substituents and linker architectures can alter the activity profile of the AZM core against strains carrying *erm* and *mef* resistance determinants [77].

**Figure 11:**
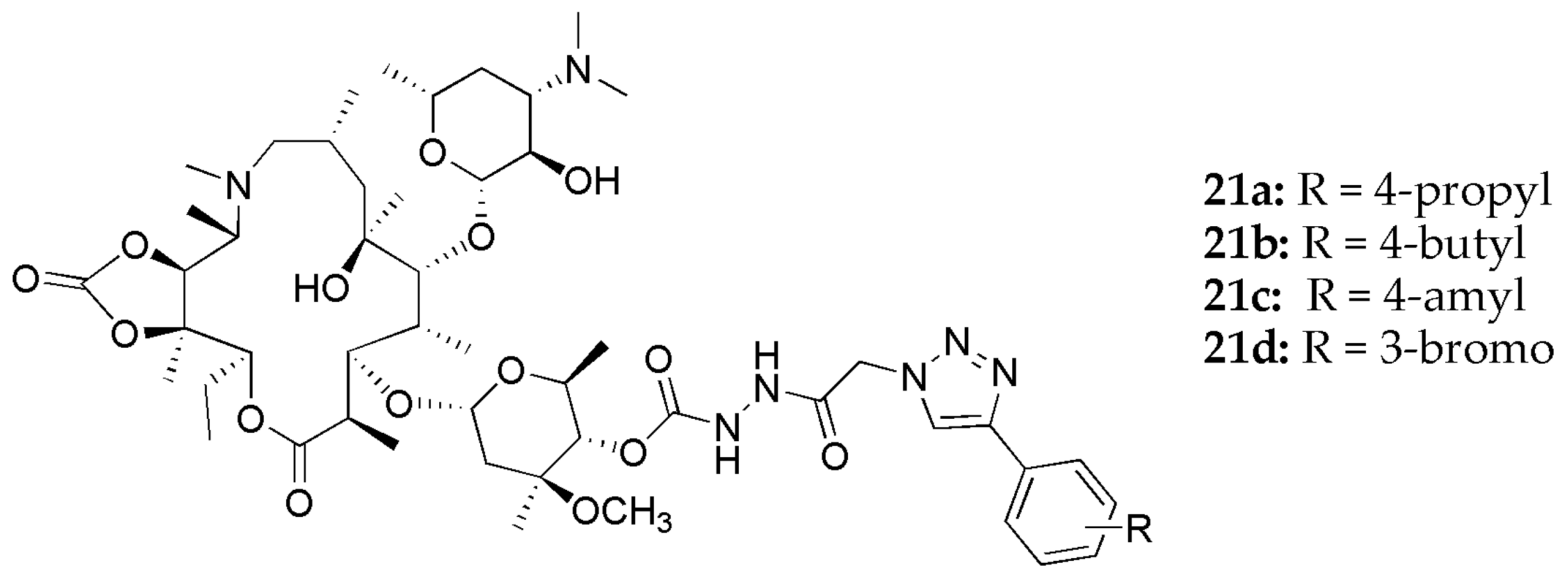
Chemical structures of 11,12-cyclic carbonate azithromycin derivatives **21a**–**d** bearing 4″-O-aralkylacetylhydrazine substituents [81].

Yan et al. synthesized 11, 12-cyclic carbonate 4″-O-aralkylacetylhydrazineacyl AZM derivatives **21a**–**d** (Figure 11), and evaluated their antibacterial activity against selected bacterial strains [81]. The derivatives were prepared by first protecting AZM at the 2′-hydroxyl group with Ac_2_O/Et_3_N, followed by formation of the 11,12-cyclic carbonate 4″-O-acylimidazolide using CDI/Et_3_N. The resulting intermediate was converted to a hydrazide, acylated with chloroacetyl chloride, and subjected to nucleophilic substitution with substituted phenols, followed by deprotection. For compounds **21a**–**d**, the chloroacetyl intermediate was converted to an azide using NaN_3_ and subsequently coupled with substituted phenylacetylenes via CuSO_4_/sodium ascorbate-catalyzed click chemistry, affording the 4″-O-(4-phenyl-triazole-1-acetyl)hydrazide AZM derivatives. Compound **21d** showed an MIC of 2 µg/mL against *S. aureus* ATCC 25923 (Table 10). Compounds **21a**–**c** showed MICs of 4 µg/mL against penicillin-resistant *S. aureus* ATCC 31007, compared with >128 µg/mL for the control drugs. Against methicillin-resistant *S. aureus* ATCC 43300, compounds **21a**–**d** showed MIC values of 4, 2, 2, and 4 µg/mL, respectively. Compounds **21b** and **21c** also showed MIC values of 2–4 µg/mL against resistant *S. aureus* strains for which the control drugs had MIC values >128 µg/mL. These findings indicate that introduction of an arylalkylacetylhydrazine side chain at the 4″ position together with a cyclic carbonate at the 11,12 positions can enhance antibacterial activity against selected resistant *S. aureus* strains. Molecular modelling suggested an interaction between the 4″-O-acetylhydrazineacyl side chain and nucleotides in the peptidyl-transferase-center region; however, this structural model does not by itself establish a causal contribution to antibacterial activity [81].

**Figure 12:**
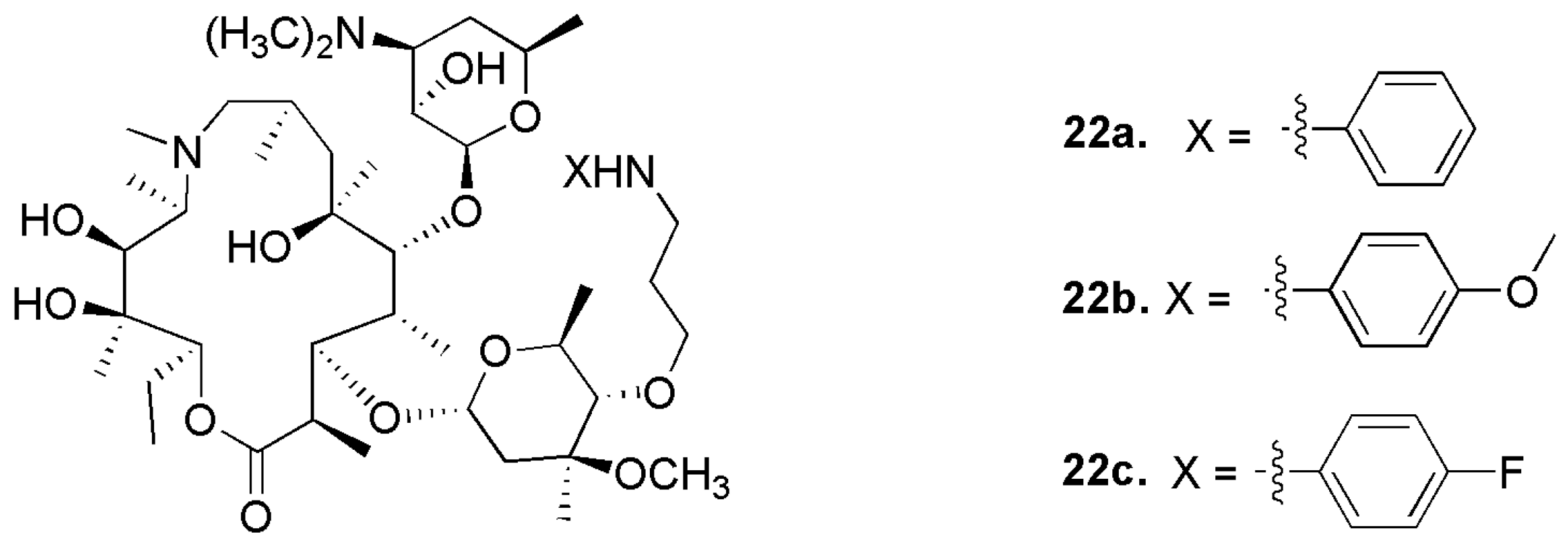
Chemical structures of compounds **22a**–**c** [82].

Jednačak and colleagues synthesized anti-infective macrozones and evaluated their antibacterial activity and structure–activity relationships [82]. Systematic variation in thiosemicarbazone side chains and coupling at 4″, 3, and 9a positions of AZM derivatives resulted in a novel class of bacterial ribosome inhibitors. Compared with AZM, the 4″-aminopropyl-macrozones **22a**–**c** (Figure 12) showed improved activity against *S. pneumoniae* and efflux-resistant *S. aureus*, with MICs ranging from 4 to 8 µg/mL (Table 11). Compounds **22a**–**c** also showed activity against *E. faecalis*, with an MIC of 1 µg/mL compared with 8 µg/mL for AZM. The SAR indicated that antibacterial activity was strongly influenced by the position of the thiosemicarbazone side chain. The presence of polar hydroxyl or ether oxygen atoms at the terminal region of the thiosemicarbazone arm was associated with lower MIC values, suggesting that polar and hydrogen-bonding interactions may contribute to ribosomal recognition. The C-4″-substituted macrozones showed activity against a range of susceptible and efflux-resistant Gram-positive bacteria, as well as *E. coli*. Their activity was also associated with cationic and zwitterionic character, hydrogen-bonding capacity, and extended π-electron delocalization involving the thiosemicarbazone moiety. These findings provide a basis for further medicinal-chemistry studies aimed at developing macrolide anti-infective agents with activity against resistant bacteria [82].

As summarized in Table 12, various structural modification and molecular hybridization strategies have been used to optimize the antibacterial activity of AZM. These approaches involve modifications at selected positions of the macrolide scaffold and the incorporation of additional pharmacophoric groups to improve antibacterial potency, broaden the spectrum of activity, and potentially overcome specific resistance mechanisms. The resulting SARs highlight the importance of modification site, substituent properties, and hybrid architecture in determining antibacterial efficacy. However, the effects of these modifications remain dependent on the chemical structure of each derivative, the bacterial strain tested, and its resistance phenotype, emphasizing the need for systematic comparative evaluation under standardized experimental conditions.

**Table 12:** Consolidated quantitative SAR evidence for AZM derivatives evaluated against resistant bacterial strains.

Modification Site/Strategy	Compounds	Representative Bacterial Strain and Resistance Phenotype	Assay Method	Derivative MIC (µg/mL)	AZM MIC (µg/mL),	AZM-Relative Fold Change *	Comparator	Mechanistic Evidence
C-4″	**2a**–**2e**, **3**	*S. pneumoniae* B0326 (M; efflux)	Broth microdilution	**2a** 2; **2b** < 0.125; **2c** 0.25; **2d** 0.25; **2e** < 0.125; **3** < 0.125	4	**2a** 2×; **2b** > 32×; **2c** 16×; **2d** 16×; **2e** > 32×; **3** > 32×	AZM	Proposed dual/expanded ribosomal interaction; evidence in review is mechanistic interpretation, not direct target assay.
C-3 O	**4**	Clinical *E. faecium*; *S. aureus* ATCC43300 and PR (resistant isolates)	Broth microdilution	*E. faecium* = 8; *S. aureus* 43300 = 16; *S. aureus* PR = 16	256; >256; >256	32×; >16×; >16×	AZM	Docking to 23S rRNA A752 plus time/concentration-dependent bactericidal kinetics; docking remains a hypothesis.
O4 glucose/sugar region	**5**–**7**	*M. avium* B-1657 (A2059C); *M. intracellulare* B-1639 (A2058G)	MIC determination; method not stated in manuscript	B-1657: **5** = 1, **6** = 4, **7** = 16; B-1639: **5** = 8, **6** = 64, **7** = 64	AZM > 128 for both	B-1657: >128×, >32×, >8×; B-1639: >16×, >2×, >2×	TEL: 4 (B-1657); 32 (B-1639)	SAR evidence linking O4 substituent position to activity; no direct target-engagement assay reported.
AZM–tedizolid	**8**, **9**	*S. aureus*-resistant panel (*erm*-mediated, L4/L22 and 23S rRNA mutant strains)	Broth microdilution reported in subsequent source; manuscript gives panel-level MIC only	**8** = 0.5; **9** = 0.25 (panel value as reported)	AZM 0.5–32 (range; not strain-matched)	Not calculable from a single matched AZM MIC; bounds from reported range would be 2–128× but are not a matched-strain fold change	Tedizolid; linezolid	Cryo-EM structures of hybrid–ribosome complexes; nine retained activity across several resistance mechanisms; human cytosolic/mitochondrial protein-synthesis assays reported.
C-11 + C-4″	**11**–**13**	*S. pneumoniae* B1 (*erm*); A22072 (*mef*); AB11 (*erm*+*mef*)	Standard dilution/MIC assay (original study)	**11**–**13**: B1 0.25; A22072 = 0.03; AB11 = 2	128; 4; 256	512×; 133×; 128×	Clarithromycin: 64; 4; 128	Strong quantitative resistance-breaking SAR; same terminal 3,5-dinitrophenyl motif; no direct target assay reported.
AZM–CLM/AZM–MNZ	**14a**–**g**, **15a**–**g**	*E. coli* ΔtolC *perm*CL-*erm*C (inducible macrolide resistance model)	Broth microdilution; CLSI	Representative: **14a** = 12.5; **14f** = 25; **15b** = 6.3; **15f =** 12.5	3.1	0.25×; 0.124×; 0.49×; 0.25× (no resistance-breaking improvement)	CLM, MNZ, ERY	Premature ribosome stalling proposed; resistance-model testing directly evaluates inducible ermC context. No direct structural target evidence in manuscript.
Thiosemicarbazone stereoisomers	**16a**–**b**, **17a**–**b**	*S. pneumoniae* B0326 (M); *S. aureus* B0331 (M); B0330 (cMLS)	Broth microdilution; CLSI	B0326: **16a =** 8, **16b** = 32, **17a** = 16, **17b** >64; B0331: 16,16, 8,16	B0326 16; B0331 > 64	B0326: 2×, 0.5×, 1×, <0.25×; B0331: >4×, >4×, >8×, >4×	None reported	Stereochemical dependence supported by comparative MICs and NMR/MS/modeling; no direct target assay.
C-4″ heterocyclic	**18a**–**g**,**19a**–**g**,**20a**–**g**	*S. pneumoniae* B-24 (*met*-positive); B-1 (*erm*-positive)	MIC assay; exact method not stated in manuscript	Quantitative resistant-strain MICs not reproduced in manuscript table	AZM comparator not reproduced for these strains	Not calculable from manuscript table	None reported	Retention against mef/erm-associated resistance is reported; mechanistic interpretation remains inferential.
4″-O hydrazine + 11,12-carbonate	**21a**–**d**	*S. aureus* ATCC31007 (penicillin-resistant); ATCC43300 (MRSA)	MIC assay; exact method not stated in manuscript	ATCC31007: **21a**–**c** = 4; ATCC43300: **21a** = 4, **21b** = 2, **21c** = 2, **21d** = 4	>128 for both resistant strains	ATCC31007: >32×; ATCC43300: >32×, >64×, >64×, >32×	Chloramphenicol	Molecular modelling suggested PTC-region nucleotide interaction; not causal proof.
C-4″ aminopropyl thiosemicarbazone	**22a**–**c**	*S. pneumoniae* B0652 (M); *S. aureus* B0331 (M)	Broth microdilution; CLSI	B0652: **22a** ≤ 0.125, **22b** ≤ 0.125, **22c** = 0.25; B0331: 4,8,4	8; >64	B0652: ≥64×, ≥64×, 32×; B0331: >16×, >8×, >16×	None reported	Polar/hydrogen-bonding and cationic/zwitterionic features associated with activity.

* Fold change is calculated as AZM MIC/derivative MIC under the matched condition. Values are bounded when the reported MIC is censored (e.g., >128 or <0.125). “Not calculable” is used where the manuscript reports an AZM range rather than a strain-matched AZM value. NR, not reported. M, macrolide-efflux phenotype; cMLS, constitutive macrolide–lincosamide–streptogramin B resistance; *erm*, erythromycin ribosomal methylation determinant; mef/met, macrolide efflux determinant; MRSA, methicillin-resistant *Staphylococcus aureus*; PTC, peptidyl-transferase center. Data source note: Quantitative MIC values and compound numbering are consolidated from Table 2, Table 3, Table 4, Table 5, Table 6, Table 7, Table 8, Table 9, Table 10 and Table 11 and the corresponding study descriptions in the manuscript.

## 5. Structure–Activity Relationships

The antibacterial activity of AZM derivatives is significantly influenced by the position, nature, and physicochemical properties of the introduced substituents. The studies summarized in this review indicate that structural modification can improve activity against resistant bacteria, but the effect is highly dependent on the modification site, side-chain architecture, linker composition, and overall molecular properties, as discussed in the following sections. Importantly, increased activity against resistant organisms is not necessarily accompanied by improved activity against susceptible strains, emphasizing the need to evaluate both absolute antibacterial potency and resistance-breaking capacity.

### 5.1. Influence of the Modification Site

The AZM scaffold contains several chemically accessible positions, including C-3, C-4″, C-9a, C-11, and C-12, which provide opportunities for structural optimization. The available data indicate that biological activity is highly dependent on the position of substitution. Modifications at C-3 and C-4″ have generated derivatives with improved activity against selected resistant Gram-positive organisms, whereas simultaneous modification of C-4″ and the C-11/C-12 region has produced derivatives with particularly enhanced activity against resistant *S.aureus* [68,75,77,81,82]. The importance of substitution position is particularly evident in thiosemicarbazone-containing AZM derivatives, where systematic variation in the attachment site at the 4″-, 3″-, and 9a-positions resulted in markedly different antibacterial profiles. The reported SAR indicated that the position of the thiosemicarbazone side chain is a major determinant of activity, with 4″-substituted macrozones generally displaying the most favorable antibacterial profiles [82].

### 5.2. C-3 Substitution and the Effect of Side-Chain Length

Modification at the C-3 position provides an effective approach for introducing additional pharmacophoric elements without completely disrupting the AZM macrocyclic framework. A representative example is compound **4**, a 3-O-substituted azalide bearing a 1,2,3-triazole-containing side chain. Compound 4 showed improved activity relative to AZM against several resistant organisms, including *E. faecium* and resistant *S. aureus*. Against these strains, compound **4** displayed MIC values of 8–16 µg/mL compared with AZM values of 256 µg/mL or greater, corresponding to a 16–32-fold improvement.

An important SAR observation was the effect of the alkyl-chain length attached to the triazole moiety. Compound **4**, which contained the longest alkyl substituent, a pentyl group, demonstrated the most potent activity within the series against several resistant strains. Molecular docking suggested that the pentyl group extends toward the A752 region of the bacterial ribosome, potentially providing additional interactions beyond those made by the parent AZM scaffold. Although this modelling provides a possible explanation for the improved activity, the proposed interaction should be regarded as a mechanistic hypothesis rather than definitive evidence of the binding contribution of the pentyl group.

These findings suggest that controlled extension of the C-3 side chain can improve antibacterial activity by increasing interactions within the ribosomal binding environment. However, the effect should not be generalized to all alkyl-chain lengths because excessive hydrophobicity or molecular size may adversely affect solubility, permeability, and pharmacokinetic properties.

### 5.3. C-3 Substitution and Side-Chain Length

Modification at the C-3 position provides an opportunity to introduce additional pharmacophoric groups while retaining the core AZM scaffold. Qin et al. reported a 3-O-substituted 15-membered azalide containing a 1,2,3-triazole side chain (compound **4**). The derivative showed improved activity compared with AZM against several resistant organisms, including *E. faecium* and resistant *S. aureus*. Against these strains, compound **4** displayed MIC values of 8–16 µg/mL compared with AZM values of 256 µg/mL or greater, corresponding to a 16–32-fold improvement [71].

An important SAR observation was the influence of alkyl-chain length on activity. The pentyl-substituted triazole derivative demonstrated the most favorable activity within the reported series. Molecular modelling suggested that the pentyl group could extend toward the A752 region of the bacterial ribosome, potentially providing additional interactions with the target [71]. Thus, controlled extension of the C-3 side chain may improve antibacterial activity by facilitating additional interactions within the ribosomal binding environment.

### 5.4. C-4″ Modification and Thiosemicarbazone-Containing Side Chains

C-4″ modification has emerged as one of the most productive approaches for improving the antibacterial activity of AZM derivatives. Grgičević et al. reported thiosemicarbazone-based AZM conjugates, or macrozones, that displayed enhanced activity against resistant bacteria [68]. The derivatives showed excellent activity against *S. pneumoniae*, with activity against the efflux-resistant M-phenotype strain being up to 32-fold greater than AZM. Several derivatives also demonstrated activity against resistant *S. aureus*, against which AZM showed little activity. However, their activity against susceptible *S. aureus* was generally lower than that of AZM, demonstrating that structural modification may preferentially improve activity against resistant phenotypes rather than universally increase potency [68].

Further SAR analysis of the macrozones showed that side-chain position and terminal functionality strongly influenced antibacterial activity. The presence of polar hydroxyl or ether oxygen atoms at the terminal region of the thiosemicarbazone side chain was associated with lower MIC values, suggesting that hydrogen-bonding interactions may contribute to improved ribosomal recognition. In addition, the antibacterial activity was favorably influenced by greater cationic or zwitterionic character, hydrogen-bonding capacity, and extended π-electron delocalization within the thiosemicarbazone moiety [82].

These findings suggest that the incorporation of an appropriately positioned polar and electronically conjugated side chain can enhance interactions with the bacterial target. The superior activity of several 4″-substituted macrozones further indicates that C-4″ represents a particularly favorable region for introducing additional antibacterial pharmacophores [68,82].

### 5.5. Heterocyclic Substitution

The incorporation of nitrogen-containing heterocycles into the AZM scaffold provides another strategy for modifying antibacterial activity. Martynov et al. investigated 4″-modified AZM derivatives containing nitrogen-containing heterocycles and reported that selected derivatives retained activity against *S. pneumoniae* strains carrying *mef*- and *erm*-associated resistance mechanisms [77]. These findings indicate that modification of the 4″-position can alter the interaction profile of the AZM scaffold sufficiently to restore antibacterial activity against strains in which conventional macrolides are compromised by efflux or ribosomal target modification.

The nature and architecture of the heterocyclic substituent are therefore important SAR determinants. The manuscript reports that different heterocycles and linker architectures produced different antibacterial profiles, emphasizing that the heterocycle cannot be considered independently from the connecting side chain. These findings support the concept that appropriate spatial positioning of an additional heterocycle may generate new interactions with the ribosome or other bacterial targets and thereby reduce susceptibility to established resistance mechanisms [77].

### 5.6. C-11 and C-12 Modifications

Modification of the C-11 and C-12 regions has also generated AZM derivatives with substantially improved antibacterial activity. Li et al. reported 11,4″-disubstituted AZM analogues containing a C-11 aryl-carbamoyl group and extended C-4″ side chains [75]. Compounds **11**–**13** demonstrated excellent activity against susceptible *S. pneumoniae* and *S. aureus* and, importantly, showed markedly improved activity against erythromycin-resistant *S. pneumoniae*. The reported derivatives were 128–512-fold more potent than AZM against selected resistant strains [75].

The strong activity observed for these disubstituted derivatives suggests that multisite modification can generate complementary interactions that are not achieved through modification of a single position. In particular, combining a C-11 aryl-carbamoyl group with an extended C-4″ side chain appears to provide a favorable structural arrangement for maintaining antibacterial activity against resistant organisms [75].

A related strategy involved modification of the C-11/C-12 region through formation of a cyclic carbonate. Yan et al. reported 11,12-cyclic carbonate AZM derivatives bearing a 4″-O-aralkylacetylhydrazineacyl side chain [81]. Compounds **21a**–**d** showed improved activity against resistant *S. aureus*. Compounds **21a**–**c** displayed MIC values of 4 µg/mL against penicillin-resistant *S. aureus*, whereas the control drugs exhibited MIC values greater than 128 µg/mL. Compounds **21b** and **21c** also demonstrated MIC values of 2 µg/mL against MRSA [81].

These results suggest that combining C-11/C-12 conformational modification with C-4″ side-chain extension can substantially enhance antibacterial activity against resistant *S. aureus*. Molecular modelling further suggested that interactions between the 4″-O-acetylhydrazineacyl side chain and nucleotides in the peptidyl-transferase-center region may contribute to the observed activity [81].

### 5.7. Modification of the Sugar Region

Modification of the sugar moieties also influences AZM activity. Isozaki et al. investigated derivatives **6** and **7** containing meta-substituted benzyl groups at the O4 position of the glucose moiety, together with triazole-containing structural features [72]. These derivatives displayed improved activity against macrolide-resistant *Mycobacterium avium* compared with AZM, although activity against *M. intracellulare* remained limited. Compound **6**, containing a 3-pyridyl group, showed an MIC of 4 µg/mL against macrolide-resistant *M. avium*, comparable to telithromycin. Compound **7** also showed improved activity against wild-type *M. avium* and *M. intracellulare*, suggesting that the position of the triazole-containing substituent contributes to activity [72].

These observations indicate that sugar-region modification can influence both target recognition and antibacterial activity, particularly against non-tuberculous mycobacteria. However, the relatively high MIC values observed against some resistant strains also demonstrate that modification of the sugar region alone may not be sufficient to overcome all macrolide-resistance mechanisms [72].

### 5.8. Molecular Hybridization and Linker Effects

Molecular hybridization represents an important strategy for incorporating additional antibacterial pharmacophores into the AZM scaffold. The antibacterial activity of AZM hybrids demonstrates that hybridization does not necessarily produce synergistic activity. The identity of the second pharmacophore, attachment site, linker length, flexibility, and overall molecular architecture all influence biological activity.

AZM–tedizolid hybrids provide an important example. Compounds **8** and **9** retained activity against several macrolide- and oxazolidinone-resistant bacterial strains. Compound **9** was comparable to or more active than linezolid against the tested strains and retained activity against strains carrying resistance mechanisms affecting macrolides and oxazolidinones [74].

The findings indicate that linker architecture is an important SAR determinant. A linker must provide sufficient flexibility to position the second pharmacophore appropriately while avoiding excessive molecular size or conformational freedom. The AZM–tedizolid hybrids demonstrate how rational positioning of two pharmacophores can generate molecules capable of interacting with bacterial ribosomes in ways that differ from their individual parent antibiotics [74].

In contrast, AZM–chloramphenicol and AZM–metronidazole hybrids generally demonstrated lower activity than expected from the parent compounds, although differences in linker structure affected antibacterial potency [76]. The manuscript reports that selected flexible linkers improved activity and that some metronidazole-containing hybrids were slightly more active than corresponding chloramphenicol hybrids [76].

These observations emphasize that increasing molecular complexity does not automatically improve antibacterial activity. Rather, the linker must preserve appropriate molecular conformation, target accessibility, and bacterial penetration.

### 5.9. Polarity, Ionization and Hydrogen-Bonding Capacity

The balance between polarity, ionization, and hydrophobicity is another important SAR determinant. In the macrozone series, incorporation of polar hydroxyl and ether functionalities was associated with improved activity, while greater cationic or zwitterionic character and hydrogen-bonding capacity were also considered favorable [82].

Nevertheless, increasing polarity does not necessarily improve activity against Gram-negative organisms. Some AZM derivatives showed weaker activity against Gram-negative bacteria despite substantial improvements against Gram-positive resistant strains. This suggests that outer-membrane permeability and intracellular accumulation remain important limitations. For example, macrozone derivatives showed only moderate activity against *H. influenzae*, while some other modified derivatives demonstrated relatively high MIC values against Gram-negative organisms [68,82].

Thus, future AZM optimization should aim to achieve an appropriate balance between ribosomal binding, ionization, hydrophobicity, molecular size, and bacterial permeability, rather than optimizing any single physicochemical parameter.

### 5.10. Mammalian Translation and Mitochondrial Toxicity: A Translational SAR Framework

The antibacterial SAR of AZM derivatives cannot be separated from the possibility of host-ribosome and mitochondrial liabilities. Mitochondrial ribosomes retain bacterial evolutionary features, making inhibition of mitochondrial protein synthesis a mechanistically plausible off-target liability for antibiotics that inhibit bacterial translation. Experimental work has reported mitochondrial effects of azithromycin itself, including reduced mitochondrial membrane potential, mitochondrial structural changes, reactive oxygen species generation, and dose-dependent inhibition of mammalian cell proliferation in human epithelial cells and fibroblasts [83]. These observations do not establish that every AZM derivative is intrinsically mitochondrial-toxic, but they provide a biologically relevant safety mechanism that should be considered when expanding the scaffold.

The reviewed C-3 and C-11 series illustrate an important evidence gap. Compound **4**, reported by Qin et al. [71], showed substantially improved activity against selected resistant bacterial strains, including 16- to >32-fold lower MIC values than AZM in some comparisons. However, the study did not report mammalian cytotoxicity, CC50 values, or mitochondrial translation data. Consequently, the reported antibacterial improvement cannot be separated from the possibility of altered mammalian exposure or host–cell translation liability. In the absence of these measurements, the compound should be regarded as an antibacterial SAR observation rather than as a demonstrated improvement in therapeutic potential.

The same concern applies to the C-11/C-4″ compounds **11**–**13** reported by Li et al. [75]. These compounds produced striking fold-improvements against selected erythromycin-resistant *S. pneumoniae* strains, including 512-, 133-, and 128-fold improvements relative to AZM. Nevertheless, the magnitude of these MIC changes does not establish a corresponding improvement in therapeutic index. The cited study does not provide a matched mammalian CC50-versus-MIC analysis or sufficient mitochondrial translation data to determine whether the additional C-11/C-4″ functionality improves bacterial selectivity or simply changes the biological activity of a substantially more complex molecule. Thus, these large fold-improvements should not be interpreted as evidence of a clinically meaningful therapeutic advantage without accompanying safety and mechanistic data.

More direct evidence is available for the AZM–tedizolid hybrid series. The cited cryo-EM study evaluated mammalian protein synthesis and showed that the hybrids altered cytosolic and mitochondrial protein synthesis differently from the parent drugs [74]. In the original study, one hybrid displayed reduced inhibition of mitochondrial protein synthesis relative to tedizolid, whereas another retained mitochondrial effects and showed additional effects on cytosolic protein synthesis and cell viability at higher concentrations. This demonstrates that hybridization can either attenuate or preserve host translation liabilities depending on linker and pharmacophore architecture; it should therefore be treated as a structure–toxicity relationship to be optimized rather than as an inherent safety advantage [74].

For the nitroaromatic/dinitrophenyl and metronidazole-containing derivatives reviewed here, the absence of systematic mammalian CC_50_, mitochondrial translation, and therapeutic-index data is a substantive translational limitation. The 3,5-dinitrophenyl-containing C-11/C-4″ derivatives are particularly notable because their antibacterial potency is high, yet the available study does not establish whether the added aromatic/nitro functionality changes mammalian exposure or toxicity [75]. Similarly, the AZM–metronidazole hybrids showed reduced antibacterial potency in several panels, but their mammalian safety window was not established in the cited antibacterial study [76]. A more informative future SAR should therefore report MIC, mammalian CC50 or IC50, and, where relevant, mitochondrial translation inhibition in the same experimental framework. A simple selectivity index (e.g., CC_50_/MIC) would provide a more meaningful measure of developability than antibacterial potency alone.

Taken together, the available evidence supports a cautious conclusion: structural expansion at C-3 or C-11 can improve antibacterial activity in selected resistance backgrounds, but there is currently insufficient comparative evidence to conclude that these modifications reduce mitochondrial or cytosolic translation inhibition. The most defensible medicinal-chemistry objective is therefore not maximal structural elaboration, but separation of bacterial activity from mammalian translation liability through balanced control of molecular size, charge, lipophilicity, intracellular distribution, and target selectivity.

### 5.11. Gram-Negative Permeability, Efflux and Physicochemical Constraints

The reviewed data reveal a recurring Gram-negative activity cliff that is better understood as a permeability–efflux–target-engagement problem than as a simple failure of ribosomal binding. The bacterial ribosomal target is highly conserved across Gram-positive and Gram-negative organisms, yet many expanded AZM derivatives have MICs of >32–64 µg/mL against *E. coli* or related Gram-negative organisms. For example, the AZM–chloramphenicol/metronidazole hybrids frequently showed MICs of 32 to >32 µg/mL against *E. coli*, the 4″-modified heterocyclic series was predominantly >32 µg/mL, and the 4″-aminopropyl macrozone series reached 32–64 µg/mL against *E. coli* [76,77,82]. These observations argue against treating the Gram-negative deficit as a universal consequence of poor target affinity.

A unified framework is therefore: (i) outer-membrane entry, (ii) intracellular accumulation and efflux, and (iii) ribosomal target engagement. AZM-like macrolides already occupy an unfavorable physicochemical space for Gram-negative penetration because of their large molecular size and amphiphilic/lipophilic character. Prior macrolide studies show that the Gram-negative outer membrane is an effective permeability barrier to azithromycin and related macrolides, while modern synthetic macrolide programmes demonstrate that reducing lipophilicity, introducing appropriately positioned basic amines, and tuning molecular weight and charge can improve permeability and efflux avoidance [24]. This framework explains why simply adding a larger heterocycle or a second antibiotic pharmacophore can improve Gram-positive resistance profiles yet simultaneously worsen Gram-negative activity: the modification may increase molecular volume, hydrophobic surface, conformational complexity, or polarity without creating a favorable route across the outer membrane.

The reviewed studies, however, generally infer reduced penetration from MIC values rather than directly measuring the underlying barrier. For this reason, statements such as “reduced cellular penetration” should be presented as plausible explanations rather than demonstrated mechanisms unless uptake, permeability, or efflux experiments were performed. A rigorous diagnostic panel would compare wild-type and outer-membrane-permeabilized strains, isogenic efflux-knockout or efflux-overexpression strains, and, where possible, intracellular accumulation measurements. In the macrolide field, the use of hyperpermeable and efflux-deficient *E. coli* strains has provided a practical way to distinguish permeability from efflux effects; this type of experimental decomposition is particularly valuable for large AZM hybrids [24].

Accordingly, structural expansion should be viewed as a trade-off. Additional pharmacophores may create new bacterial interactions or evade a resistance determinant, but they may also increase the permeability penalty. The most informative SAR is therefore not “larger substituent = stronger activity,” but “additional functionality is beneficial only when the resulting molecule remains within a physicochemical space compatible with bacterial entry, intracellular retention, and target engagement.” This interpretation is consistent with the reviewed AZM–chloramphenicol/metronidazole series, in which flexible linkers altered activity but did not rescue the pronounced Gram-negative MIC penalty [76], and with the heterocyclic series, in which substantial Gram-positive activity coexisted with MICs >32 µg/mL against *E. coli* [77]. Table 13 summarizes the unified SAR and physicochemical framework linking structural modifications of AZM derivatives to antibacterial activity, permeability, intracellular accumulation, and overall developability.

### 5.12. Interpreting Docking and Structural Evidence

The molecular-docking and structural observations reviewed in this manuscript should be classified according to their evidentiary strength. Docking can generate plausible poses and identify candidate interactions, but docking scores and visual proximity do not establish binding affinity or prove that a particular contact is responsible for antibacterial activity. Accordingly, the pentyl–A752 interaction proposed for compound **4** should be described as a hypothesis [71]. Similarly, the modelled interaction of the 4″-O-acetylhydrazineacyl side chain with the peptidyl-transferase-center region should be treated as a structural rationale rather than a demonstrated energetic contribution [81].

Cryo-EM provides stronger structural evidence than docking because it directly observes ligand-associated density in the ribosome complex, but even a resolved pose does not by itself establish which individual contacts dominate affinity or activity. The Yeon et al. study is therefore especially informative because structural positioning was integrated with microbiological testing and mammalian protein-synthesis assays [74]. The appropriate language throughout this review should distinguish “observed density/pose,” “consistent with a proposed interaction,” and “experimentally demonstrated contribution.” Where no ITC, SPR, competition-binding, mutational, biochemical, or equivalent orthogonal validation was reported, atomic-level interaction claims should remain explicitly provisional.

This distinction also helps separate genuine pharmacophoric advances from synthetic noise. A modification should be considered mechanistically informative when a structural change is reproducibly associated with activity across related analogues and, ideally, is supported by orthogonal evidence such as resistance-genotype profiling, biochemical target assays, competition binding, structural determination, or cellular accumulation measurements. By contrast, an isolated MIC improvement accompanied only by a docking pose should be regarded as an SAR observation requiring validation, not as proof of a new pharmacophore or a new binding mechanism.

### 5.13. Overall SAR Trends

Collectively, the reviewed studies support a more selective SAR framework. C-3 modification can provide a route for adding functionality to the AZM scaffold, but the benefit of side-chain extension is context-dependent and may be offset by permeability or safety penalties [71]. C-4″ modification appears repeatedly useful for resistance-oriented optimization, particularly when the appended group is appropriately positioned and the linker balances polarity, charge, and conformational freedom [68,77,82]. C-11/C-12 modification can produce large improvements against selected erythromycin-resistant organisms when combined with complementary C-4″ substitution, but the magnitude of the MIC improvement should not be equated with a proven therapeutic advantage [75,81]. Molecular hybridization can provide genuine resistance-bypassing potential when the two pharmacophores are geometrically compatible with adjacent ribosomal sites, as illustrated by the structurally characterized AZM–tedizolid series [74]; however, hybridization can also reduce activity through impaired accumulation or unfavorable linker geometry [76].

The central medicinal-chemistry lesson is therefore that resistance circumvention requires simultaneous optimization of target engagement, resistance mechanism avoidance, bacterial permeability, intracellular accumulation, and host safety. The most credible leads are not necessarily the derivatives with the largest MIC fold-improvements or the most elaborate structures, but those for which antibacterial potency is retained at low absolute MIC values while mammalian translation, mitochondrial function, permeability, and pharmacokinetic liabilities remain controlled.

Collectively, the studies reviewed demonstrate several important SAR principles. First, the position of substitution is critical, with C-3, C-4″, C-11, and C-12 representing particularly useful sites for structural optimization [68,75,77,81,82]. Second, side-chain length and composition can significantly influence antibacterial activity, as demonstrated by the C-3 triazole-containing derivatives [71]. Third, linker architecture is an important determinant of hybrid activity, as demonstrated by the AZM–tedizolid and AZM–chloramphenicol/metronidazole series [74,76]. Fourth, polar and hydrogen-bonding functionalities can improve activity in selected derivative classes, particularly the thiosemicarbazone macrozones [82]. Finally, the most promising modifications are those that retain the fundamental AZM scaffold while introducing additional interactions capable of improving activity against resistant bacteria.

Importantly, fold-improvement values should be interpreted together with absolute MIC values. For example, compound **4** showed a 16–32-fold improvement over AZM against selected resistant bacteria but its MIC values remained 8–16 µg/mL [71]. Therefore, resistance-breaking activity should not by itself be interpreted as evidence of high antibacterial potency. Future optimization should seek derivatives that combine low absolute MIC values, activity against clinically relevant resistant strains, favorable physicochemical and pharmacokinetic properties, and acceptable safety profiles.

## 6. Conclusions

The increasing prevalence of antimicrobial resistance has significantly reduced the clinical effectiveness of macrolide antibiotics such as AZM, highlighting the need for structural modification of AZM to enhance its antibacterial potential. This review shows that the versatile 15-membered azalide scaffold of AZM provides several chemically accessible sites, including C-3, C-4″, C-9a, C-11, and C-12, as well as the desosamine and cladinose moieties, for rational structural modification.

The studies discussed demonstrate that side-chain modification, heterocyclic incorporation, sugar modification, and molecular hybridization can substantially alter the antibacterial activity. However, the evidence also exposes a persistent limitation in the current AZM-derivative literature: antibacterial potency is frequently characterized more rigorously than bacterial exposure, mammalian selectivity, and mechanism of action. Several derivatives show impressive fold-improvements in MIC against selected resistant organisms, yet these improvements are not consistently accompanied by mammalian CC50/IC50 measurements, mitochondrial and cytosolic translation assays, intracellular-accumulation studies, permeability/efflux decomposition, or direct target-engagement experiments.

This distinction is critical. A large reduction in MIC, particularly when calculated against a highly resistant parent control, does not by itself demonstrate a therapeutic breakthrough. For example, the C-11/C-4″ derivatives reported by Li et al. showed very large fold-improvements against selected resistant strains, but the absence of a matched mammalian therapeutic-window analysis prevents those values from being interpreted as evidence of improved clinical selectivity. Similarly, the C-3 derivative reported by Qin et al. demonstrated resistance-associated MIC improvements, but without mammalian CC50, mitochondrial translation, or uptake data, its broader therapeutic significance remains unresolved.

The field should therefore move beyond “more potent” and “more structurally complex” as primary indicators of progress. Future AZM-derivative studies should establish, as far as experimentally feasible, four linked properties: (i) low absolute MIC values against clinically relevant resistant pathogens; (ii) a demonstrable mammalian selectivity index or therapeutic window; (iii) evidence that the compound reaches and accumulates within the relevant bacterial compartment while overcoming permeability and efflux barriers; and (iv) direct or orthogonal evidence supporting the proposed molecular mechanism and target engagement. Mitochondrial and cytosolic translation profiling should be incorporated where host translation liability is mechanistically plausible.

Accordingly, an isolated fold-improvement in an MIC table should be regarded as an SAR observation, not a therapeutic breakthrough, unless it is supported by complementary safety, exposure, and mechanistic evidence. The most valuable AZM derivatives will not necessarily be those with the largest substituents or the largest MIC fold-changes, but those that demonstrate a reproducible separation between bacterial antibacterial activity and mammalian toxicity while maintaining appropriate permeability, intracellular accumulation, pharmacokinetic properties, and mechanistic selectivity. The future of AZM medicinal chemistry should therefore shift from the accumulation of increasingly elaborate analogues toward mechanism-, exposure-, safety-, and developability-driven optimization. Such an approach will help distinguish genuine resistance-breaking pharmacological advances from compounds whose apparent superiority is limited to isolated antibacterial potency measurements.

## Figures and Tables

**Table 1:** Mechanisms of AZM resistance in different bacterial strains.

Bacterial Strains	Mechanisms of Resistance	Refs.
*Escherichia coli*	Emergence of *mef*(C)-*mph*(G) and *erm*(42) genes and distinct *mph*(A) operon structures.	[27]
*Neisseria gonorrhoeae*	Mutations in multiple copies of the 23S rRNA genes and their regulatory genes. Efflux**:** *Mtr*R mutations causing efflux pump overexpression.	[28,29]
*Enterobacteriaceae*	Target mutations: 23S rRNA and ribosomal protein mutations. Methylation: *erm*-, *cfr*-, and *rlmA*-mediated methylation. Decreased uptake: Efflux pumps and outer membrane alterations. Macrolide modification: Esterases and phosphotransferases. Peptide-mediated resistance: Target protection by resistance peptides.	[30]
*Mycoplasma genitalium*	23S rRNA mutations, particularly A2058/A2059.	[31]
*Streptococcus pneumoniae*	23S rRNA/L4 mutations and alterations in glutamine metabolism or rRNA maturation. *erm*(B) methylation; *mef*(A/E) efflux; ribosomal mutations.	[32]
*Campylobacter jejuni*	23S rRNA mutations, especially A2075G; efflux mechanisms.	[33,34,35]
*Shigella* spp.	*mph*(A)-mediated macrolide inactivation; conjugative plasmids; efflux.	[36]
*Streptococcus pyogenes*	Expansion of *emm12*-ST36 GAS carrying *erm*(B) and *tet*(M) resistance genes.	[37]
*Treponema pallidum* ssp. *pallidum*	23S rRNA A2058G/A2059G mutations.	[38]
*Legionella pneumophila*	Mutations in the *lpeAB* efflux pump gene and in genes encoding 23S rRNA or the L4 and L22 ribosomal proteins.	[39]
*Pseudomonas aeruginosa*	Efflux pump-mediated resistance during biofilm formation in *P. aeruginosa*. 23S rRNA mutations associated with azithromycin resistance.	[40,41]
*Chlamydia trachomatis*	Mutations in *rplD* (L4), the 23S rRNA peptidyl transferase region, and the non-conserved L22 region.	[42]

**Table 2:** In vitro antibacterial activity of thiosemicarbazone-based azithromycin conjugates **2a**–**2e** and **3** compared with azithromycin [68].

Strains	MIC (µg/mL)						
	2a	2b	2c	2d	2e	3	AZM
*S. pneumoniae* ATCC 49619	<0.125	<0.125	<0.125	<0.125	<0.125	<0.125	<0.125
*S. pneumoniae* B0326 (M phenotype)	2	<0.125	0.25	0.25	<0.125	<0.125	4
*S. pyogenes* B0542	0.25	<0.125	0.25	0.5	0.25	0.25	<0.125
*S. aureus* ATCC 29213	8	8	4	4	4	>64	1
*S. aureus* B0331 (M)	64	8	8	4	4	>64	>64
*E. faecalis* ATCC 29212	8	1	1	1	1	<0.125	4
*H. influenzae* ATCC 49247	ND	16	16	16	8	64	2

Footnote: MIC, minimum inhibitory concentration; AZM, azithromycin; ND, not determined. M phenotype, macrolide efflux phenotype.

**Table 3:** In vitro antibacterial activity of the 3-O-substituted 15-membered azalide derivative **4** [71] compared with azithromycin.

Strains	MIC (µg/mL)	
	Compound 4	AZM
*S. aureus* ATCC25923	8	≤0.25
*E. faecalis* ATCC29212	4	4
*B. subtilis* ATCC9372	8	≤0.25
Clinical isolate of *E. faecium*	8	256
*S. aureus* ATCC43300	16	>256
*S. aureus* PR	16	>256
*K. pneumoniae* ATCC10031	32	2

Footnote: MIC, minimum inhibitory concentration; AZM, azithromycin.

**Table 4:** In vitro antibacterial potency of compounds **5**–**7** comparable to AZM and TEL [72,73].

Strains	MIC (µg/mL)				
	5	6	7	AZM	TEL
*M. avium* subsp. *avium* ATCC25291 (wild-type)	0.25	0.125	0.0625	1	1
*M. avium* subsp. *avium* B-1657 (A2059C)	1	4	16	>128	4
*M. intracellulare* JCM6384 (wild-type)	0.125	0.0312	0.0625	0.125	0.125
*M. intracellulare* B-1639 (A2058G)	8	64	64	>128	32

**Table 5:** Antibacterial activity of compounds **8** and **9** against *S. aureus* [74].

Strain	MIC (µg/mL)			
	8	9	AZM	TDZ
*S. aureus*	0.5	0.25	2	0.25

**Table 6:** In vitro bacterial activity of compounds **11**–**13** compared with precursor **10** [75].

Strains	MIC (µg/mL)					
	10	11	12	13	AZM	CAM
*S. pneumoniae* ATCC49619	0.12	0.03	0.03	0.03	0.03	0.03
*S. pneumoniae* B1	1	0.25	0.25	0.25	128	64
*S. pneumoniae* A22072	0.12	0.03	0.03	0.03	4	4
*S. pneumoniae* AB11	8	2	2	2	256	128
*S. aureus* ATCC25923	2	0.25	0.25	0.12	0.25	0.12
*S. typhimurium* TA102	1	0.5	0.5	0.25	0.25	0.25

**Table 7:** In vitro antibacterial activity of azithromycin–chloramphenicol and azithromycin–metronidazole hybrid derivatives **14a**–**g** and **15a**–**g** compared with the parent drugs [76].

	**AZM–Chloramphenicol Hybrids MIC (µg/mL)**								
**Strains**	**14a**	**14b**	**14c**	**14d**	**14e**	**14f**	**14g**	**CLM**	**AZM**
*S. pneumoniae* ATCC 49619	0.5	0.25	0.25	0.25	0.5	0.125	0.5	4	0.06
*S. aureus* ATCC 29213	32	32	32	16	32	16	>32	8	0.5
*E. coli* ATCC 25922	32	>32	>32	>32	>32	>32	>32	8	2
	**AZM** **–** **Metronidazole Hybrids MIC (µg/mL)**								
**Strains**	**15a**	**15b**	**15c**	**15d**	**15e**	**15f**	**15g**	**MNZ**	**AZM**
*S. pneumoniae* ATCC 49619	0.25	0.125	0.125	0.125	0.125	0.06	0.25	>32	0.06
*S. agalactiae* 1Cp	1–2	1	1–2	1	0.5	0.5	1	>32	0.015
*S. aureus* ATCC 29213	8─16	8	16	4	8	4	32	>32	0.5
*C. sporogenes* ATCC 19404	0.25	4	8	8	4	4	16	8	4
*P. acnes* 55	4	4	2	2	0.25	8	4	>32	<0.06

Footnote: MIC, minimum inhibitory concentration; AZM, azithromycin; CLM, chloramphenicol; MNZ, metronidazole.

**Table 8:** In vitro antibacterial activity of azithromycin–thiosemicarbazone macrozones **16a** and **17a** and their related derivatives (**16b**, **17b**) compared with AZM.

Strains	MIC (µg/mL)				
	16a	16b	17a	17b	AZM
*S. pneumoniae* B0652 (eryS)	8	16	2	16	≤0.125
*S. pneumoniae* B0326 (M)	8	32	16	>64	16
*S. pneumoniae* B0633 (cMLS)	64	64	64	>64	>64
*S. pyogenes* B0542 (eryS)	2	16	4	32	≤0.125
*S. pyogenes* B0545 (M)	8	16	16	64	>64
*S. pyogenes* B0544 (cMLS)	32	32	64	64	>64
*S. aureus* ATCC29213 (eryS)	8	16	8	>32	1
*S. aureus* B0331 (M)	16	16	8	16	>64
*S. aureus* B0330 (cMLS)	32	8	16	16	>64
*E. faecalis* ATCC29212	8	16	32	>64	8
*E. coli* ATCC25922	>64	>64	>64	>64	>64

Footnote: MIC, minimum inhibitory concentration; AZM, azithromycin; eryS, erythromycin-sensitive; M, macrolide efflux phenotype; cMLS, constitutive macrolide–lincosamide–streptogramin B resistance; ATCC, American Type Culture Collection.

**Table 9:** Antibacterial activity of AZM derivatives bearing diverse heterocyclic and aromatic substituents.

	MIC (µg/mL)			
	*S. pneumoniae* ATCC 49619	*S. aureus* ATCC 29213	*E. faecalis* ATCC 29212	*E. coli* ATCC 25922
AZM	0.06	1	8	8
**18i**	16	32	32	>32
**19i**	>32	>32	>32	>32
**20i**	>32	>32	>32	>32
**18a**	0.5	4	4	32
**19a**	0.25	4	2	32
**20a**	0.25	4	4	32
**18b**	0.25	>32	16	>32
**19b**	0.125	32	16	>32
**20b**	0.125	>32	16	>32
**18c**	16	32	32	>32
**19c**	16	32	16	>32
**20c**	16	32	16	>32
**18d**	0.125	8	4	>32
**19d**	0.06	8	8	>32
**20d**	0.06	8	4	>32
**18e**	0.125	4	4	32
**19e**	0.03	1	1	32
**20e**	0.06	2	4	32
**18f**	0.125	4	4	>32
**19f**	0.06	2	0.5	>32
**20f**	0.03	4	4	>32
**18g**	0.03	8	8	32
**19g**	0.015	4	0.5	>32
**20g**	0.06	8	8	32

**Table 10:** In vitro antibacterial activity of azithromycin derivatives **21a**–**d** against selected *S. aureus* strains compared with azithromycin and comparator antibiotics.

Strains	MIC(µg/mL)					
	21a	21b	21c	21d	AZM	CAM
*S. aureus* ATCC25923	4	4	4	2	≤0.25	≤0.25
*S. aureus* ATCC31007	4	4	4	8	>128	>128
*S. aureus* ATCC43300	4	2	2	4	>128	>128

Footnote: MIC, minimum inhibitory concentration; AZM, azithromycin; CAM, comparator antibiotic as reported in the original study; ATCC, American Type Culture Collection.

**Table 11:** In vitro antibacterial activity of 4″-aminopropyl thiosemicarbazone-based azithromycin macrozones **22a**–**c** compared with azithromycin [82].

Strains	MIC (µg/mL)			
	22a	22b	22c	AZM
*S. pneumoniae* B0652 (M)	≤0.125	≤0.125	0.25	8
*S. aureus* ATCC29213 (eryS)	4	8	4	1
*S. aureus* B0331 (M)	4	8	4	>64
*E. faecalis* ATCC 29212	1	1	1	8
*E. coli* ATCC 25922	64	64	32	8

Footnote: MIC, minimum inhibitory concentration; AZM, azithromycin; eryS, erythromycin-sensitive; M, macrolide efflux phenotype; ATCC, American Type Culture Collection.

**Table 13:** Unified SAR and physicochemical framework for interpreting AZM derivative activity and developability.

Structural Feature/Change	Potential Antibacterial Benefit	Likely Limiting Factor	What the Current Literature Supports and What Remains Unproven	Priority Validation
C-3 side-chain extension/triazole	May restore activity against selected resistant Gram-positive strains; activity varies with chain length	Increased size/hydrophobicity may reduce solubility, penetration or increase host exposure	Improved activity against selected resistant Gram-positive strains is demonstrated. However, the study does not establish a therapeutic advantage because mammalian CC50/cytotoxicity, mitochondrial translation, and intracellular accumulation were not reported. The proposed A752 interaction remains a docking hypothesis [71].	Binding assay or structural validation; mammalian CC_50_ and mitochondrial translation; uptake studies
C-4″ thiosemicarbazone/heterocycle	Can retain activity against selected *mef*/*erm*-resistant strains and add functionality	Large aromatic/linker additions may impair Gram-negative entry and alter exposure	Comparative MIC data support an SAR involving polarity, hydrogen bonding and charge, but these physicochemical interpretations are not equivalent to direct mechanistic proof. Intracellular accumulation, target engagement and mammalian selectivity remain insufficiently established [68,77,82].	Efflux/permeability panel; intracellular accumulation; direct target/translation assays
C-11/C-12 + C-4″ disubstitution	Large improvements against selected erythromycin-resistant *S. pneumoniae* and *S. aureus*	High molecular complexity and limited safety data; possible permeability penalty	Large MIC improvements against selected erythromycin-resistant strains are experimentally demonstrated. However, the reported 512-, 133-, and 128-fold improvements do not establish an improved therapeutic index because matched mammalian safety and mitochondrial translation data are lacking. No direct target-engagement assay was reported [75].	CC_50_/IC_50_ vs MIC; mitochondrial translation; PK and permeability profiling
AZM–tedizolid hybridization	Can engage adjacent ribosomal regions and bypass several resistance mechanisms	Linker geometry can misposition the second pharmacophore; mitochondrial liability may persist	This series provides stronger mechanistic evidence because structural data were integrated with antibacterial and mammalian protein-synthesis measurements. Nevertheless, the data demonstrate that hybridization can retain or alter mammalian translation liabilities; it should therefore be optimized rather than assumed to confer safety [74].	Quantitative binding/translation assays and broader mammalian safety profiling
AZM–CLM/AZM–MNZ hybrids	May alter resistance phenotype and provide dual pharmacology	Frequently reduced Gram-negative activity; increased size/linker burden	Linker-dependent changes in antibacterial activity are demonstrated, but several hybrids show reduced activity, particularly against Gram-negative organisms. Reduced penetration remains an interpretation unless directly supported by uptake/permeability measurements. Mammalian safety windows remain insufficiently characterized [76].	Outer-membrane/efflux decomposition and mammalian safety window

## Data Availability

No new data were created or analyzed in this study. Data sharing is not applicable to this article.
